# DIPG Harbors Alterations Targetable by MEK Inhibitors, with Acquired Resistance Mechanisms Overcome by Combinatorial Inhibition

**DOI:** 10.1158/2159-8290.CD-20-0930

**Published:** 2022-03-08

**Authors:** Elisa Izquierdo, Diana M. Carvalho, Alan Mackay, Sara Temelso, Jessica K.R. Boult, Giulia Pericoli, Elisabet Fernandez, Molina Das, Valeria Molinari, Yura Grabovska, Rebecca F. Rogers, Maria Antonietta Ajmone-Cat, Paula Z. Proszek, Mark Stubbs, Sarita Depani, Patricia O'Hare, Lu Yu, Georgia Roumelioti, Jyoti S. Choudhary, Matthew Clarke, Amy R. Fairchild, Thomas S. Jacques, Richard G. Grundy, Lisa Howell, Susan Picton, Jenny Adamski, Shaun Wilson, Juliet C. Gray, Bassel Zebian, Lynley V. Marshall, Fernando Carceller, Jacques Grill, Maria Vinci, Simon P. Robinson, Michael Hubank, Darren Hargrave, Chris Jones

**Affiliations:** 1 Division of Molecular Pathology, Institute of Cancer Research, London, United Kingdom.; 2 Division of Radiotherapy and Imaging, The Institute of Cancer Research, London, United Kingdom.; 3 Department of Haematology/Oncology, Gene and Cell Therapy, Bambino Gesù Children's Hospital-IRCCS, Rome, Italy.; 4 National Centre for Drug Research and Evaluation, Istituto Superiore di Sanità, Rome, Italy.; 5 Molecular Diagnostics, Royal Marsden Hospital NHS Trust, Sutton, United Kingdom.; 6 Division of Cancer Therapeutics, Institute of Cancer Research, London, United Kingdom.; 7 Department of Haematology and Oncology, Great Ormond Street Hospital for Children NHS Foundation Trust, London, United Kingdom.; 8 Division of Cancer Biology, Institute of Cancer Research, London, United Kingdom.; 9 UCL Great Ormond Street Institute for Child Health, London, United Kingdom.; 10 Children's Brain Tumour Research Centre, School of Medicine, University of Nottingham, Nottingham, United Kingdom.; 11 Alder Hey Children's NHS Foundation Trust, Liverpool, United Kingdom.; 12 Leeds Children's Hospital, Leeds, United Kingdom.; 13 Birmingham Women's and Children's Hospital, Birmingham, United Kingdom.; 14 Oxford University Hospitals NHS Foundation Trust, Oxford, United Kingdom.; 15 Centre for Cancer Immunology, University of Southampton, Southampton, United Kingdom.; 16 Department of Neurosurgery, Kings College Hospital NHS Trust, London, United Kingdom.; 17 Division of Clinical Studies, The Institute of Cancer Research, London, United Kingdom.; 18 Children & Young People's Unit, Royal Marsden Hospital NHS Trust, Sutton, United Kingdom.; 19 Department of Pediatric and Adolescent Oncology and INSERM Unit U891, Team “Genomics and Oncogenesis of Pediatric Brain Tumors,” Gustave Roussy and University Paris-Saclay, Villejuif, France.

## Abstract

The survival of children with diffuse intrinsic pontine glioma (DIPG) remains dismal, with new treatments desperately needed. In a prospective biopsy-stratified clinical trial, we combined detailed molecular profiling and drug screening in newly established patient-derived models *in vitro* and *in vivo*. We identified *in vitro* sensitivity to MEK inhibitors in DIPGs harboring MAPK pathway alterations, but treatment of patient-derived xenograft models and a patient at relapse failed to elicit a significant response. We generated trametinib-resistant clones in a *BRAF*^G469V^ model through continuous drug exposure and identified acquired mutations in *MEK1/2* with sustained pathway upregulation. These cells showed hallmarks of mesenchymal transition and expression signatures overlapping with inherently trametinib-insensitive patient-derived cells, predicting sensitivity to dasatinib. Combined trametinib and dasatinib showed highly synergistic effects *in vitro* and on *ex vivo* brain slices. We highlight the MAPK pathway as a therapeutic target in DIPG and show the importance of parallel resistance modeling and combinatorial treatments for meaningful clinical translation.

**Significance:**

We report alterations in the MAPK pathway in DIPGs to confer initial sensitivity to targeted MEK inhibition. We further identify for the first time the mechanism of resistance to single-agent targeted therapy in these tumors and suggest a novel combinatorial treatment strategy to overcome it in the clinic.

*This article is highlighted in the In This Issue feature, p. 587*

## Abstract (precis)

A proportion of diffuse intrinsic pontine gliomas (DIPG) harbor alterations that predict sensitivity to MEK inhibition; however, resistance can quickly occur via MEK1/2 mutation that can be overcome with addition of dasatinib, providing a therapeutic option.

## Introduction

The MAPK pathway plays an important role in signal transduction regulating cell proliferation, differentiation, and cell death (1). Dysregulation of the MAPK signaling pathway is implicated in a wide range of cancers as a result of genetic and epigenetic alterations. In adults, *BRAF*-mutated tumors include 60% of melanomas, 60% of thyroid cancers, 15% of colorectal cancers, and 5% to 8% of non–small cell lung cancers, with the most prevalent mutation being *BRAF*^V600E^ (2). In addition, non-V600E mutations have been identified to be oncogenic and can be classified as one of three types based on their effect on BRAF activity (3, 4).

MAPK pathway alterations are commonly found in childhood cancer, particularly brain tumors, and especially low- and high-grade gliomas (5). These include pilocytic astrocytoma (*KIAA1549:BRAF* tandem duplication, *RAF* fusions, *NF1*, *FGFR1*, *BRAF*^V600E^; refs. 6, 7), mixed glioneuronal tumors (*FGFR1*, *BRAF*^V600E^, *KIAA1549:BRAF*; refs. 8–10), pleomorphic xanthoastrocytomas (*BRAF*^V600E^; refs. 11, 12), infant pediatric high-grade glioma (*pHGG; NTRK1/2/3*, *ROS1*, *ALK*, *MET* fusions; refs. 13–15), non-brainstem pHGG (*FGFR1*, *NF1*, *BRAF*^V600E^, *NTRK2*^ITD^, *MET*; refs. 13, 16–18), and diffuse intrinsic pontine glioma (DIPG; *PIK3R1*, *NF1*; refs. 13, 19). For the latter, with a median survival of 9 to 12 months (20), they represent an unexplored option in a tumor type in desperate need of novel treatment strategies (21).

Although targeted agents against the MAPK pathway have become an important initial success story in the field of precision oncology, they are frequently associated with the emergence of resistance and treatment failure (22). Therapy-induced resistance can occur from the acquisition of *de novo* mutations (23, 24) or from expansion of rare preexisting resistant cells, also called “persister clones” (25–27). The use of inhibitors such as those targeting BRAF (vemurafenib, dabrafenib) and MEK (trametinib, selumetinib, cobimetinib) in *BRAF*^V600E^-positive melanoma resulted in a moderate success of targeted therapies by showing tumor shrinkage and improving patient survival (28–30). However, durable responses were limited due to resistance to single agents often mediated by reactivation of MAPK through amplification or splice variants in *BRAF*, mutations in the upstream oncogene *NRAS* or the downstream kinase *MEK1* (*MAP2K1*), and PI3K–PTEN–AKT upregulation, among others (24, 31–34). This led to the combination of BRAF and MEK inhibitors in clinical trials, which showed a significant improvement in overall survival compared with single-agent therapy, and subsequently FDA-approved treatment for advanced *BRAF*^V600E^-positive melanoma (35, 36). However, acquired resistance was observed in patients under BRAF and MEK inhibitors, with the identification of *MEK1* and *MEK2* (*MAP2K2*) mutations as two of the main mechanisms (31, 37–40).

Despite their clinical availability for treating children with brain tumors harboring targetable alterations (41, 42), BRAF and MEK inhibitors have thus far not been explored in DIPG. As part of a coclinical trial of patients with DIPG from the United Kingdom enrolled in BIOMEDE, an adaptive, international, phase II clinical trial in newly diagnosed DIPG (NCT02233049), we have generated patient-derived *in vitro* and *in vivo* models subjected to integrated drug screening and molecular profiling. We identify specific genetic dependencies associated with response to trametinib *in vitro*, explore mechanisms of acquired resistance that emerge to single-agent targeted therapy, and present rational drug combinations that may circumvent this in future clinical trials.

## Results

### MAPK Alterations Confer *in Vitro* Sensitivity to Trametinib in DIPG Models from a Coclinical Trial

**Figure 1.**
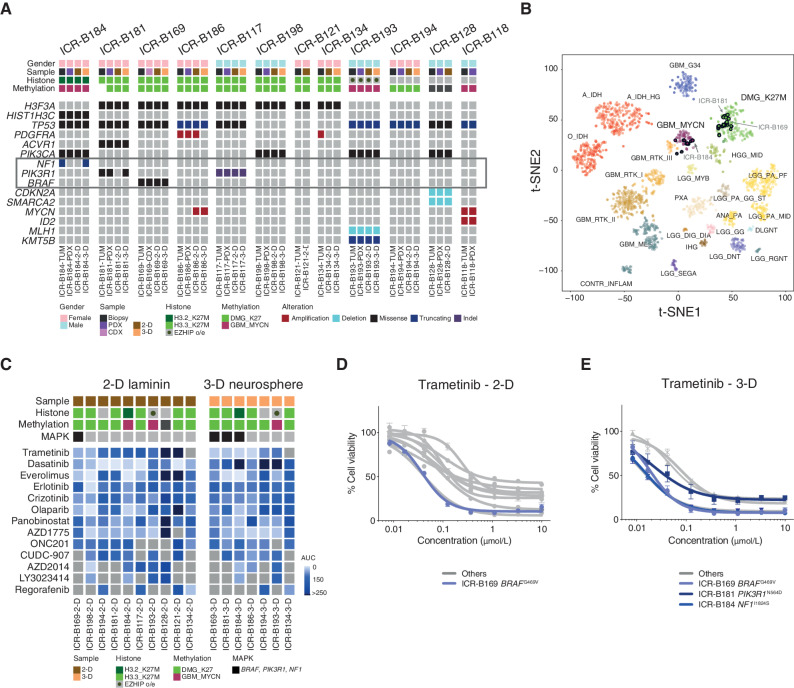
*In vitro* sensitivity to trametinib in patient-derived DIPG models. **A,** Oncoprint representation of an integrated annotation of single-nucleotide variants, DNA copy number changes, and structural variants for patient-derived models and tumor biopsy specimens. Samples are arranged in columns with genes labeled along rows. Clinicopathologic and molecular annotations are provided as bars according to the included key. **B,** The *t*-statistic–based stochastic neighbor embedding (t-SNE) projection of a combined methylation data set comprising the *in vitro* models (circled) plus a reference set of glioma subtypes (*n* = 1,766). The first two projections are plotted on the *x*- and *y*-axes, with samples represented by dots colored by subtype as labeled on the figure. **C,** Drug sensitivities in the mini-screens carried out on cells grown under 2-D and 3-D conditions, visualized by heat map of normalized AUC values. Clinicopathologic and molecular annotations are provided as bars according to the included key. **D,** Dose–response curves for the MEK inhibitor trametinib tested against patient-derived models *in vitro* grown in 2-D. **E,** Dose–response curves for the MEK inhibitor trametinib tested against patient-derived models *in vitro* grown in 3-D. Cells harboring MAPK pathway alterations are highlighted in blue. Concentration of compound is plotted on a log scale (*x*-axis) against cell viability (*y*-axis). Means plus standard errors are plotted from at least *n* = 3 experiments.

BIOMEDE (NCT02233049) was a phase II, biopsy-driven clinical trial in patients with DIPG with randomization of stratification between dasatinib, erlotinib, and everolimus. From UK patients, we undertook to generate patient-derived *in vitro* and *in vivo* models from biopsy material excess to trial inclusion. From the first 12 cases, exome sequencing was performed on the biopsy specimen itself, and panel sequencing was performed on the *in vitro* cultures established under stem cell conditions in 2 dimensions (2-D; on a laminin matrix) and 3 dimensions (3-D; as neurospheres), as well as tumors grown as orthotopic xenografts in mice (Fig. 1A). Seven cases presented with *H3F3A* (now *H3–3A*) K27M mutations, one had *HIST2H3C* (now *H3C14*) K27M, and another presented *EZHIP* overexpression by RNA sequencing, recently described as phenocopying the H3K27M mutation-driven loss of H3K27me3 (43). Of the three histone H3 wild-type cases, one harbored *MYCN/ID2* coamplification. Recognized recurrent DIPG driver mutations were largely preserved between the biopsy and models, with specific exceptions between 2-D and 3-D cultures described below, and some amplicons (*PDGFRA*, *MYCN*) discrepant between different samples from the same patient (Fig. 1A). Similarly, all models were highly concordant with their original tumors on the basis of their methylation profiles derived from Illumina 850K EPIC arrays (Fig. 1B). Although all *H3F3A*^K27M^ and *EZHIP* cases clustered with other diffuse midline glioma K27M tumors, the *HIST2H3C*^K27M^ case (ICR-B184) was most similar to ICR-B118 and other MYCN-amplified glioblastoma in the *t*-statistic–based stochastic neighbor embedding projection of the methylation array data (Fig. 1B). The H3 wild-type case ICR-B128 did not classify strongly with any known subgroup. A summary of the molecular data of the models and the tumors from which they were derived is given in Supplementary Table S1.

Seventeen established cultures from 11 patients were subjected to a drug mini-screen against a range of targeted inhibitors (*n* = 13), selected on the basis of their molecular profiles (Fig. 1C). Exploring these data for genetic dependencies, we observed a striking concordance of mutations in the MAPK pathway (*BRAF*, *NF1*, *PIK3R1*) and sensitivity to the MEK inhibitor trametinib, both in 2-D and 3-D, including the models ICR-B169 in 2-D (Fig. 1D) and ICR-B169, ICR-B181, and ICR-B184 in 3-D (Fig. 1E).

### Functional Selection of MAPK Alterations in Model Systems and Translation to the Clinic

**Figure 2.**
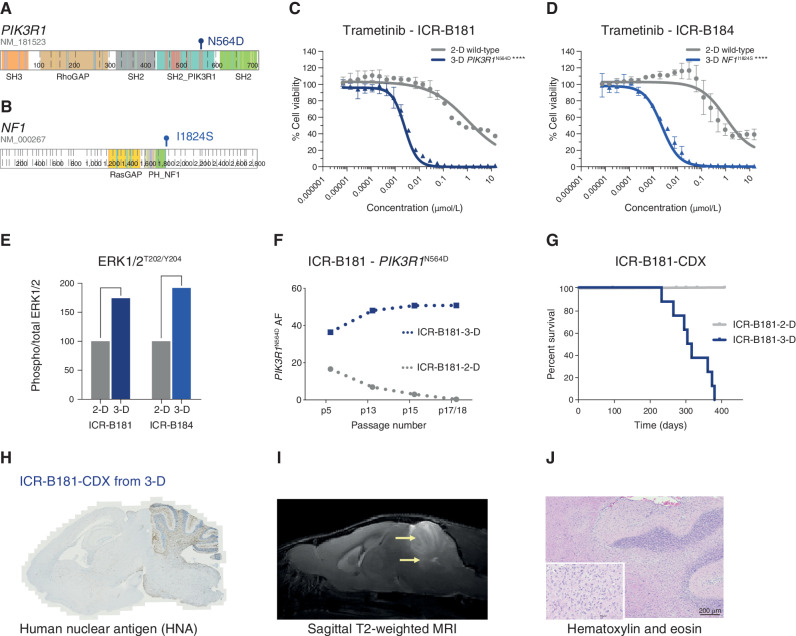
*PIK3R1* and *NF1* mutations drive the sensitivity of DIPG cells to trametinib. **A,** Cartoon representing the protein domains of *PIK3R1* showing the mutant residue for the observed hotspot N564D mutation observed in ICR-B181. **B,** Cartoon representing the protein domains of *NF1* showing the mutant residue for the observed I1824S missense mutation observed in ICR-B184. Generated in ProteinPaint (pecan.stjude.cloud/proteinpaint). **C,** Dose–response validation curves for trametinib tested against ICR-B181 cells *in vitro* grown in 3-D (*PIK3R1*^N564D^, blue) and 2-D (*PIK3R1* wild-type, gray). **D,** Dose–response curves for trametinib tested against ICR-B184 cells *in vitro* grown in 3-D (*NF1*^I1824S^, blue) and 2-D (*NF1* wild-type, gray). Concentration of compound is plotted on a log scale (*x*-axis) against cell viability (*y*-axis). Mean plus standard error are plotted from at least *n* = 3 experiments. ****, *P* < 0.0001, AUC *t* test. **E,** Bar plot of quantitative capillary phospho-protein assessment of phospho-ERK1/2^T202/Y204^, plotted as a ratio to total ERK1/2, and normalized to the 2-D (MAPK wild-type) model in each case. **F,** VAF (*y*-axis) of *PIK3R1*^N564D^ in ICR-B181 cells grown in 3-D (blue) and 2-D (gray) over time, as measured by ddPCR. Passage number of cells assessed is given on the *x*-axis. **G,** Survival curves for ICR-B181-CDX models, separated by mice implanted with cells grown as either 2-D (gray) or 3-D (blue). **H,** Anti–human nuclear antigen (HNA), staining for ICR-B181-CDX derived from cells grown in 3-D, with extensive tumor cell infiltration. Sagittal sections, counterstained with hematoxylin. **I,** Sagittal T_2_-weighted image (day 246 postimplantation) for ICR-B181-CDX derived from cells grown in 3-D, showing hyperintense tumor throughout the cerebellum and upper pons (indicated by arrow). **J,** Hematoxylin and eosin–stained section of ICR-B181–3-D CDX, showing histology consistent with high-grade glioma. Scale bar, 200 μm.

ICR-B181 harbored a subclonal [variant allele frequency (VAF) = 18%] hotspot mutation in *PIK3R1*, N564D (Fig. 2A). ICR-B184 was found to have a subclonal (VAF = 20%) frameshift mutation in *NF1* in the biopsy specimen (E78fs) that was absent from the models, which instead presented the missense mutation I1824S (Fig. 2B). In both cases, the likely MAPK-activating mutations were observed in only the 3-D cultures, not 2-D cultures, which corresponded with a marked increase in sensitivity to trametinib in the neurospheres compared with adherent cells, when validated in a full dose-response analysis [∼420-fold for ICR-B181 (Fig. 2C) and ∼460-fold for ICR-B184 (Fig. 2D), *P* < 0.0001 AUC *t* test]. Similar results were observed with the additional MEK inhibitors cobimetinib [10-fold for ICR-B181, *P* = 0.0012 AUC *t* test (Supplementary Fig. S1A) and 28-fold for ICR-B184, *P* = 0.0004 AUC *t* test (Supplementary Fig. S1B)] and binimetinib [6-fold for ICR-B181, *P* = 0.0035 AUC *t* test (Supplementary Fig. S1C) and 18-fold for ICR-B184, *P* = 0.0007 AUC *t* test (Supplementary Fig. S1D)].

Using a quantitative capillary phospho-protein platform, we observed approximately twofold higher basal levels of MAPK pathway activation (phospho-ERK1/2^T202/Y204^) in the mutant 3-D compared with wild-type 2-D cultures for both cell models (Fig. 2E). Notably, the *PIK3R1*^N564D^ mutation was present in both 2-D (36/227 reads, VAF = 16%) and 3-D (100/301 reads, VAF = 33%) cultures of ICR-B181 at passage 5, whereas it was completely absent from the laminin culture at passage 18 (0/342 reads) while presumably clonally heterozygous in the neurospheres (195/396 reads, VAF = 49%). These positive and negative selections in culture were confirmed over sequential passages by a bespoke Droplet Digital PCR (ddPCR) assay (Fig. 2F; Supplementary Table S2). By contrast, the *NF1*^I1824S^ mutation was never observed in the ICR-B184 2-D cultures (0/201 reads at passage 4). Remarkably, orthotopic engraftment of ICR-B181 2-D [p18, 20/5476 *PIK3R1*^N564D^ droplets, VAF = 0.32% (Supplementary Fig. S1E)] failed to produce tumors after 12 months, whereas implantation of ICR-B181 3-D [p17, 4919/9727 *PIK3R1*^N564D^ droplets, VAF = 50.6% (Supplementary Fig. S1F)] gave rise to 10/10 NSG mice with a median overall survival of 310 days (Fig. 2G), producing diffusely infiltrative tumors (Fig. 2H) visible on MRI (Fig. 2I) and histologically consistent with a high-grade glioma (Fig. 2J).

**Figure 3.**
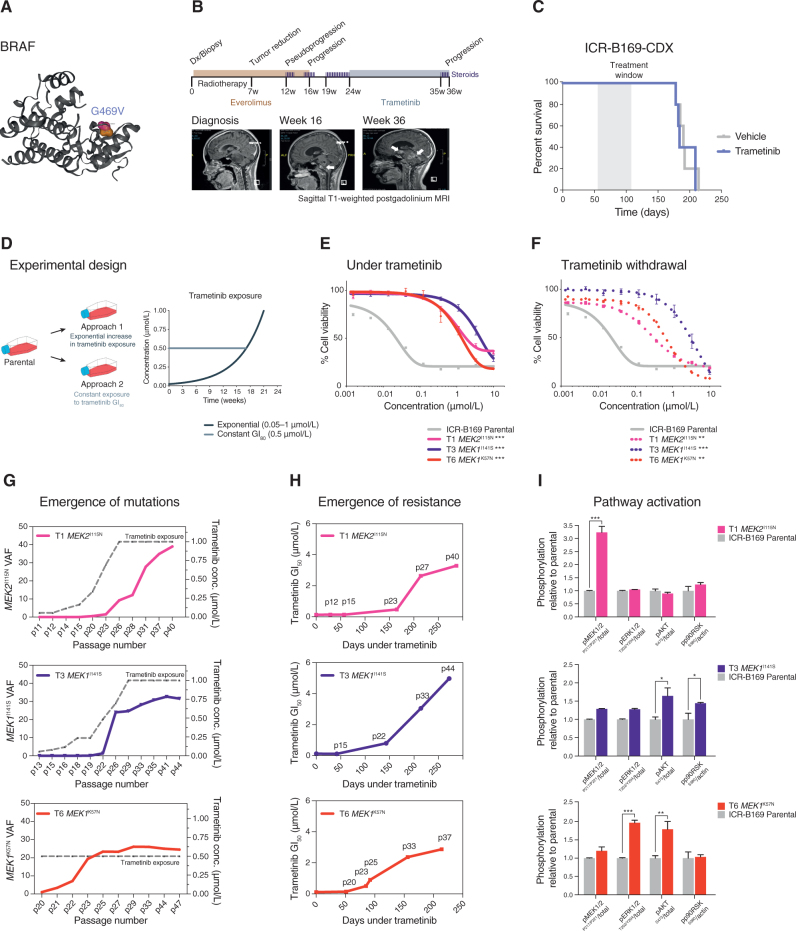
MEK1/2 mutations drive resistance to trametinib in BRAF^G469V^-driven DIPG cells. **A,** Protein structure representation of BRAF showing the mutant residue (shaded orange) for the observed G469 missense mutation observed in ICR-B169. Generated in COSMIC-3D (cancer.sanger.ac.uk/cosmic3d). **B,** Timeline of clinical experience for the child with *BRAF*^G469V^-mutant DIPG treated with trametinib at progression. Initial therapy with everolimus and radiotherapy is shaded in orange, later treatment with trametinib in blue. Sagittal T1-weighted postgadolinium MRI images are provided at diagnosis, the initial progression, and the later progression immediately prior to death from disease. Periods of steroid treatment are noted by purple lines. The tumor is highlighted with arrows. **C,** Survival curves for mice bearing ICR-B169 cell-derived orthotopic xenografts, treated with trametinib (blue), compared with vehicle-treated controls (gray). Treatment window is shaded in gray. **D,** Experimental design for the generation of cells resistant to trametinib by the continuous exposure model. Parental ICR-B169 cells are treated with either an exponentially increasing dose of inhibitor over time (approach 1) or a constant IC_80_ dose (approach 2). **E,** Dose–response curves for trametinib tested against ICR-B169 parental cells (gray) and resistant clones T1 (*MEK2*^I115N^, pink), T3 (*MEK1*^I141S^, purple), and T6 (*MEK1*^K57N^, red) after 7 to 9 months of exposure to inhibitor. **F,** Dose–response curves for trametinib tested against ICR-B169 parental cells (gray) and resistant clones (dashed lines) after 2-month withdrawal of inhibitor. Concentration of compound is plotted on a log scale (*x*-axis) against cell viability (*y*-axis). Means plus standard errors are plotted from at least *n* = 3 experiments. **, *P* < 0.001; ***, *P* < 0.001, AUC *t* test. **G,** Emergence of *MEK1/2* mutations in clones T1 (pink), T3 (purple), and T6 (red) over time, as assessed by ddPCR. The *x*-axes represent passage number; left *y*-axes are specific mutation VAFs; right *y*-axes plot the concentration of trametinib that cells were exposed to (gray dashed line). **H,** Emergence of resistance in clones over time (T1, pink; T3, purple; T6, red), plotted as days exposed to inhibitor on the *x*-axis, with passage numbers labeled. The *y*-axis is a GI_50_ value for trametinib in cells harvested at the given passage. **I,** Pathway activation in resistant clones (T1, pink; T3, purple; T6, red) assessed by a capillary electrophoresis assay and plotted as a ratio of respective phosphorylated/total protein compared to ICR-B169 parental cells (gray).

ICR-B169 (2-D and 3-D) harbored a clonal, heterozygous, noncanonical class II *BRAF* oncogenic mutation, G469V (Fig. 3A). Following feedback of our molecular and *in vitro* drug screening data, the patient was clinically treated with trametinib on a compassionate use basis (0.025 mg/kg/d once daily, the pediatric recommended phase II dose previously established in an international phase I/II clinical trial) 2 months after progression at 16 weeks on everolimus and radiotherapy. During 11 weeks on trametinib, the patient improved clinically, was able to be taken off the steroid treatments required over the previous 3 months, and showed radiologically stable disease during this time. However, an MRI at week 12 showed progression, with the appearance of new metastatic lesions within the brainstem and in the lateral ventricles, and the patient died of disease shortly afterward (Fig. 3B). In parallel, mice bearing orthotopic xenografts derived from ICR-B169 cells (3-D) were treated with trametinib, from day 55 postimplantation, 1 mg/kg orally once daily over 53 days (5 days on, 2 days off). No difference in overall survival between trametinib- and vehicle-treated animals was observed (*P* = 0.7123, log-rank test; Fig. 3C).

### MEK1/2 Mutations Drive Resistance to Trametinib in BRAF^G469V^-Driven DIPG Cells

As single-agent trametinib did not achieve the expected efficacy both *in vivo* and clinically, we sought to explore the mechanisms by which resistance to MEK inhibition may occur in DIPG and identify strategies to circumvent these. We first attempted to generate subclones of ICR-B169 cells resistant to trametinib by two continuous exposure approaches *in vitro*, the first by an exponential increase in drug concentration from the GI_50_ value over time and the second by a constant GI_80_ dose (Fig. 3D). Both experiments produced subclones with increasing resistance to trametinib, with shifts in GI_50_ values over 5 to 9 months of 64- to 167-fold with the exponential model (Supplementary Fig. S2A) and 5- to 56-fold with the constant GI_80_ approach (Supplementary Fig. S2B). To explore what might be underlying the lack of sensitivity in selected subclones, exome sequencing was carried out in the most resistant cells, compared with both the original tumor biopsy specimen and the parental ICR-B169 cells. Strikingly, all three clones were found to contain mutations in either *MEK1* (K57N or I141S) or *MEK2* (I115N; Supplementary Fig. S2C). The three models chosen for further work were 46-fold (T1, *MEK2*^I115N^), 101-fold (T3, *MEK1*^I141S^), and 33-fold (T6, *MEK1*^K57N^) more resistant to trametinib than parental cells (*P* = 0.0003–0.0008, AUC *t* test; Fig. 3E), a phenotype maintained after the inhibitor was withdrawn for 2 months (*P* = 0.0003–0.0096, AUC *t* test; Fig. 3F), as were the *MEK1/2* mutation VAFs (T1 *MEK2*^I115N^, 12%–14.6%; T3 *MEK1*^I141S^, 28.2%–30.5%; T6 *MEK1*^K57N^, 26%–24.9%). Compared with the parental cells, the resistant clones required 10- to 100-fold higher concentrations of trametinib to effectively inhibit phospho-ERK1/2, as measured by quantitative capillary phospho-protein analysis (Supplementary Fig. S2D).

To evaluate if resistance was selected from preexisting tumor subclones or was acquired *in vitro* in response to trametinib, allele-specific ddPCR assays for the *MEK1/2* mutations were conducted in both the original tumor biopsy specimen and the parental cells (Supplementary Fig. S3A). No mutant droplets were found in either sample for any of the three mutations, with an average number of wild-type droplets of 32,165 and 41,083, respectively. We also found no *MEK1/2* mutant droplets in either vehicle- or trametinib-treated cell-derived xenograft (CDX) specimens from our earlier efficacy experiment. Exome sequencing of these latter specimens confirmed the lack of *MEK1/2* mutations, but in four of five treated mice, we observed plausible variants in genes that converge on RAF/MEK signaling, including *MYBL1*, *IRAK1*, *DUSP26*, and others (Supplementary Fig. S3B), highlighted by STRING protein-interaction analysis (Supplementary Fig. S3C).

The trametinib-resistant clones were also cross-resistant to the additional MEK inhibitors cobimetinib (*P* < 0.0001–0.0004 AUC *t* test; Supplementary Fig. S4A) and binimetinib (*P* = 0.0021–0.0033 AUC *t* test; Supplementary Fig. S4B), with GI_50_ values not reached at 10 μmol/L drug. When implanted orthotopically in immune-deficient mice, they had a substantially longer latency time to tumor formation, as seen with median survival times of 225 to 435 days, compared with the 185 days of the ICR-B169 parental cells (Supplementary Fig. S4C).

The allele-specific *MEK1/2* ddPCR assays were further used to track the emergence of *MEK1/2* mutations from longitudinal passages over the course of the continuous exposure experiments. Under both experimental conditions, *MEK1/2* VAF increased in an exponential manner over time, but an earlier emergence was observed in the clone exposed to constant GI_80_ trametinib concentration (T6 *MEK1*^K57N^; Fig. 3G). This increase in *MEK1/2* mutation frequency was mirrored by the increasing GI_50_ values over sequential passages (Fig. 3H). In all three resistant models, *MEK1/2* mutations conferred an enhanced constitutive MAPK pathway activation by quantitative capillary phospho-protein analysis. This was, however, read out at different nodes in the pathway with different *MEK1*/*2* mutations, with T1 *MEK2*^I115N^ showing enhanced phospho-MEK1/2^P217/P221^ (*P* < 0.0001), T3 *MEK1*^I141S^ showing increased phospho-AKT^S473^ (*P* = 0.0166) and phospho-P90/RSK^S380^ (*P* = 0.0096), and T6 *MEK1*^K57N^ showing higher phospho-ERK1/2^P202/P204^ (*P* = 0.0006) and phospho-AKT^S473^ (*P* = 0.0074, all one-way ANOVA; Fig. 3I).

### Mechanisms of *MEK1/2* Mutation-Driven Resistance to Trametinib

To explore the mechanism by which mutations in *MEK1/2* confer resistance to trametinib in DIPG cells, we carried out gene expression profiling by RNA sequencing (RNA-seq), as well as total and phospho-proteome analysis by LC/MS-MS in our resistant clones. We identified 277, 127, and 241 differentially upregulated genes and 232, 175, and 214 downregulated genes at the transcript level in T1, T3, and T6, respectively, compared with ICR-B169 parental cells (Supplementary Fig. S5A). Of those, a total of 41 genes were shared to be upregulated and 56 genes to be downregulated among the three clones (Supplementary Fig. S5B and S5C). From the total proteome, 447, 378, and 435 proteins were differentially overexpressed and 626, 673, and 729 underexpressed in the clones (Supplementary Fig. S5D), with 137 and 225, respectively, in common between the three clones (Supplementary Fig. S5E and S5F). Phospho-proteomics identified a total of 212, 37, and 167 phospho-peptides from 124, 22, and 106 proteins with increased phosphorylation and 561, 171, and 395 phospho-peptides from 373, 117, and 221 proteins with decreased phosphorylation in T1, T3, and T6, respectively (Supplementary Fig. S5G). Of these, a total of 8 proteins with increased phosphorylation and 57 with decreased phosphorylation were shared among the three clones (Supplementary Fig. S5H and S5I).

**Figure 4.**
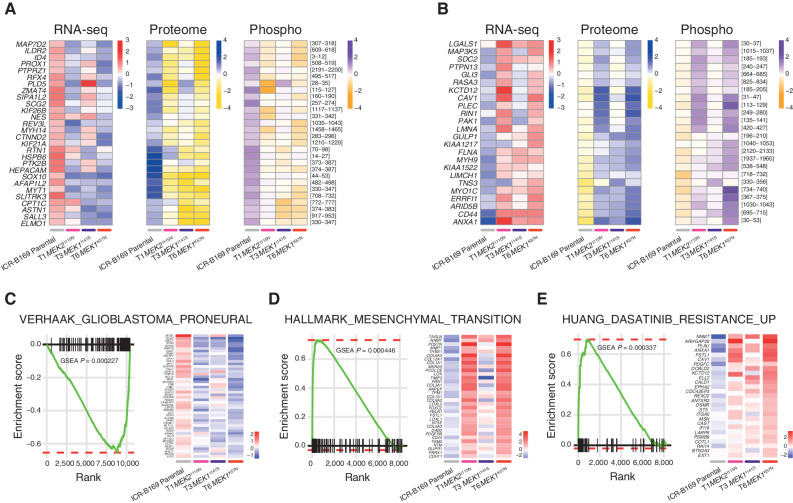
Integrated gene and protein expression profiling of trametinib-resistant DIPG cells. **A,** Coordinately downregulated genes (left), proteins (middle), and phospho-sites (right) in all three trametinib-resistant subclones of ICR-B169 *BRAF*^G469V^ cells as compared with parental. **B,** Coordinately upregulated genes (left), proteins (middle), and phospho-sites (right) in all three trametinib-resistant subclones, as compared with ICR-B169 parental (gray). T1, *MEK2*^I115N^, pink; T3, *MEK1*^I141S^, purple; T6, *MEK1*^K57N^, red. **C,** GSEA enrichment plots for the signature VERHAAK_GLIOBLASTOMA_PRONEURAL in T6 *MEK1*^K57N^ cells compared with ICR-B169 parental. The curves show the enrichment score on the *y*-axis and the rank list metric on the *x*-axis. Alongside is a heat map representation of expression of significantly differentially expressed genes in the signature in all three trametinib-resistant clones compared with parental. **D,** GSEA enrichment plots for the signature HALLMARK_EPITHELIAL_MESENCHYMAL_ TRANSITION in T6 *MEK1*^K57N^ cells compared with ICR-B169 parental. The curves show the enrichment score on the *y*-axis and the rank list metric on the *x*-axis. Alongside is a heat map representation of expression of significantly differentially expressed genes in the signature in all three trametinib-resistant clones compared with parental. **E,** GSEA enrichment plots for the signature HUANG_DASATINIB_RESISTANCE_UP in T6 *MEK1*^K57N^ cells compared with ICR-B169 parental. The curves show the enrichment score on the *y*-axis and the rank list metric on the *x*-axis. Alongside is a heat map representation of expression of significantly differentially expressed genes in the signature in all three trametinib-resistant clones compared with parental. T1, *MEK2*^I115N^, pink; T3, *MEK1*^I141S^, purple; T6, *MEK1*^K57N^, red.

The intersection of the shared most differentially upregulated (*n* = 28; Fig. 4A) or downregulated (*n* = 24; Fig. 4B) genes, proteins, and phospho-sites between any of the three clones resulted in an integrated signature of coordinately regulated signaling changes induced by *MEK1/2* mutation in response to challenge by trametinib treatment. Gene set enrichment analysis (GSEA) of this integrated signature showed a high degree of concordance between the clones (Supplementary Fig. S6A and S6B) and included activation of numerous processes involved with cytoskeleton reorganization, cell migration, cell polarization, and cell matrix remodeling, as well as depletion of neural and oligodendrocyte markers. Consequently, there was a highly significant underrepresentation of such differentiation profiles such as the VERHAAK_GLIOBLASTOMA_PRONEURAL signature in the clones (nominal *P* = 0.000446; Fig. 4C), along with an enrichment of mesenchymal gene sets such as HALLMARK_EPITHELIAL_MESENCHYMAL_TRANSITION (nominal *P* = 0.000227; Fig. 4D). Most notably, there was also a significant enrichment of a gene signature indicative of sensitivity of cancer cells lines to dasatinib, HUANG_DASATINIB_RESISTANCE_UP (nominal *P* = 0.000337; Fig. 4E, Supplementary Table S3).

### Combinatorial Approaches for Overcoming Trametinib Resistance in DIPG

**Figure 5.**
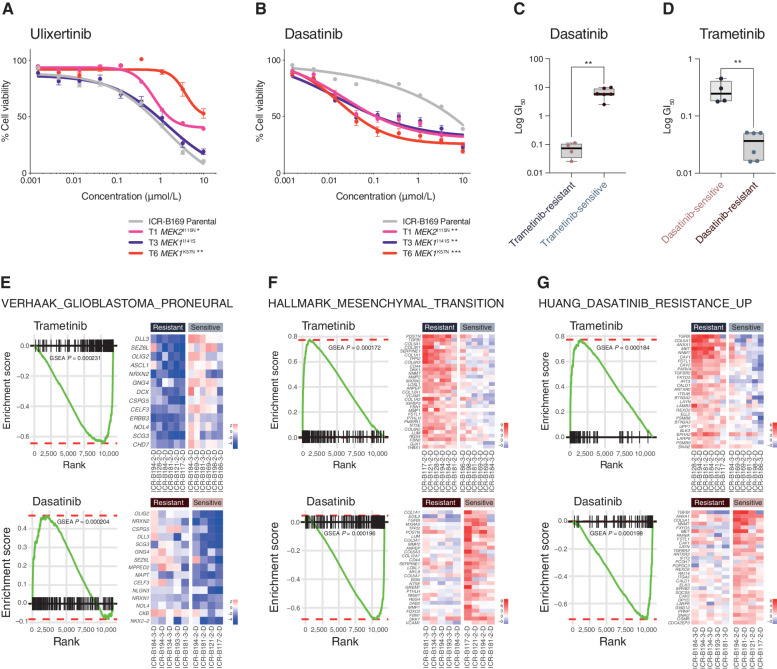
Reciprocity of drug sensitivities and gene expression signatures between trametinib and dasatinib in DIPG cells. **A,** Dose–response curves for ulixertinib tested against ICR-B169 parental cells (gray) and resistant clones T1 (*MEK2*^I115N^, pink), T3 (*MEK1*^I141S^, purple), and T6 (*MEK1*^K57N^, red). **B,** Dose-response curves for dasatinib tested against ICR-B169 parental cells (gray) and resistant clones. Concentration of compound is plotted on a log scale (*x*-axis) against cell viability (*y*-axis). Means plus standard errors are plotted from at least *n* = 3 experiments. *, *P* < 0.05; **, *P* < 0.01; ***, *P* < 0.001, AUC *t* test. **C,** Box plot of dasatinib GI_50_ values (log scale, *y*-axis) for primary patient-derived cultures separated by trametinib sensitivity status. **D,** Box plot of trametinib GI_50_ values (log scale, *y*-axis) for primary patient-derived cultures separated by dasatinib sensitivity status. **, *P* < 0.001, *t* test. **E,** GSEA enrichment plots for the signature VERHAAK_GLIOBLASTOMA_PRONEURAL in primary patient-derived cultures separated by trametinib (top) or dasatinib (bottom) sensitivity status. The curves show the enrichment score on the *y*-axis and the rank list metric on the *x*-axis. Alongside are heat map representations of expression of significantly differentially expressed genes in the signature in all trametinib- or dasatinib-resistant versus sensitive cell cultures. **F,** GSEA enrichment plots for the signature HALLMARK_EPITHELIAL_ MESENCHYMAL_TRANSITION in primary patient-derived cultures separated by trametinib (top) or dasatinib (bottom) sensitivity status. The curves show the enrichment score on the *y*-axis and the rank list metric on the *x*-axis. Alongside are heat map representations of expression of significantly differentially expressed genes in the signature in all trametinib- or dasatinib-resistant versus sensitive cell cultures. **G,** GSEA enrichment plots for the signature HUANG_DASATINIB_RESISTANCE_UP in primary patient-derived cultures separated by trametinib (top) or dasatinib (bottom) sensitivity status. The curves show the enrichment score on the *y*-axis and the rank list metric on the *x*-axis. Alongside are heat map representations of expression of significantly differentially expressed genes in the signature in all trametinib- or dasatinib-resistant versus sensitive cell cultures.

In considering opportunities for how to effectively treat MAPK-driven DIPG cells that have become resistant to MEK inhibitors, we first tested the downstream ERK inhibitor ulixertinib, as reported in other tumor types (44, 45). No differences in sensitivity were observed between T1 *MEK2*^I115N^ or T3 *MEK1*^I141S^ and the ICR-B169 parental cells, whereas T6 *MEK1*^K57N^ cells were significantly less sensitive to ulixertinib (*P* = 0.0024, AUC *t* test; Fig. 5A). By contrast, treatment with the upstream receptor tyrosine kinase (RTK) inhibitor dasatinib revealed a marked differential sensitivity in all trametinib-resistant clones, with a 68- to 174-fold shift in GI_50_ values (*P* = 0.009–0.0004, AUC *t* test; Fig. 5B). With this reciprocal potency of trametinib and dasatinib in the ICR-B169 parental cells and subclones, we next investigated whether this was true in all the patient-derived cultures we initially assessed. Cells classed as trametinib-resistant in the mini-screen were found to have significantly lower GI_50_ values for dasatinib than the trametinib-sensitive cultures (*P* = 0.0017, unpaired *t* test; Fig. 5C), while the converse was also true: dasatinib-sensitive cells had significantly higher GI_50_ values for trametinib than those classed as dasatinib-resistant (*P* = 0.0014, unpaired *t* test; Fig. 5D).

Given the similarities between a range of distinct patient-derived cultures (and their relative responses to trametinib) and the resistant clones generated from the *BRAF*^G469V^-driven ICR-B169 cells, we applied GSEA to the RNA-seq data on the panel of untreated *in vitro* models. There was substantial overlap in the significantly differentially expressed gene signatures observed in the inherently trametinib-resistant primary cultures and those seen in the *MEK1/2*-mutant resistant ICR-B169 clones (Supplementary Fig. S6C). Most strikingly, there was a highly significant underrepresentation of the VERHAAK_GLIOBLASTOMA_PRONEURAL signature in the inherently trametinib-resistant cultures (nominal *P* = 0.000231), significantly enriched in dasatinib-resistant cells (nominal *P* = 0.000204; Fig. 5E), along with the reverse pattern for mesenchymal gene sets such as HALLMARK_EPITHELIAL_MESENCHYMAL_TRANSITION (trametinib-resistant, nominal *P* = 0.000172; dasatinib- resistant, nominal *P* = 0.000196; Fig. 5F). As expected, we also observed a significant enrichment in the inherently trametinib-resistant cells, as well as underrepresentation in the dasatinib-resistant primary cultures, of the HUANG_DASATINIB_RESISTANCE_UP gene set (nominal *P* = 0.000184 and nominal *P* = 0.000198, respectively; Fig. 5G, Supplementary Table S3). As dasatinib is a multitargeted kinase inhibitor, we explored which of these may underlie the response in the trametinib-resistant cells by phospho-protein array profiling. In T6 (*MEK1*^K57N^) cells, we observed only a modest decrease in pPDGFRB^Y571^ (Supplementary Fig. S6D) and pAKT^S473^ (Supplementary Fig. S6E) in response to dasatinib at the higher doses. By contrast, there were high basal levels of pSRC^Y419^ (Supplementary Fig. S6F) and pYES1^Y426^ (Supplementary Fig. S6G), which were dramatically inhibited at the lowest doses of dasatinib, correlating with the cellular response (Supplementary Table S4). Notably, there was also a specific shift in dasatinib sensitivity in the paired ICR-B181 2-D and ICR-B181 3-D (*PIK3R*1^N546D^) cells (3-fold, *P* = 0.0398 AUC *t* test; Supplementary Fig. S6H), as well as the ICR-B184 2-D and ICR-B184 3-D (*NF1*^I1824S^) cells (7.7-fold, *P* = 0.0012 AUC *t* test; Supplementary Fig. S6I).

**Figure 6.**
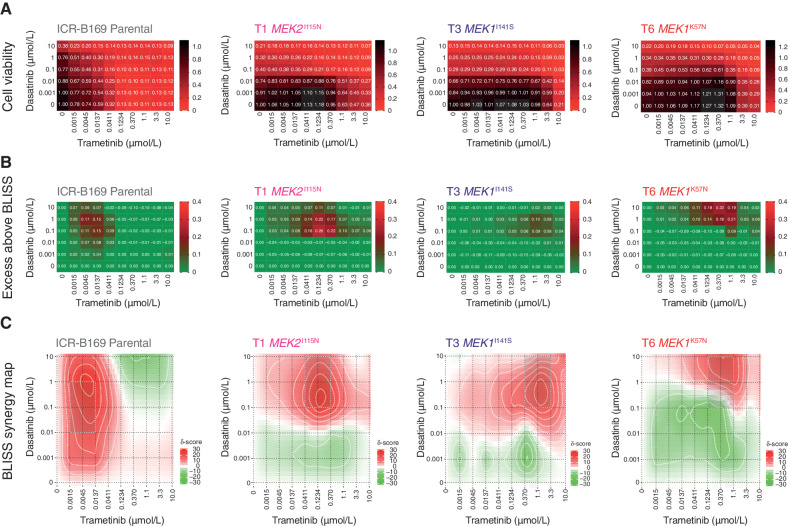
Synergy of combined dasatinib and trametinib in BRAF^G469V^-driven DIPG cells. **A,** Cell viability matrices for ICR-B169 parental (gray) and trametinib-resistant clones T1 (*MEK2*^I115N^, pink), (T3 *MEK1*^I141S^, purple), and T6 (*MEK1*^K57N^, red), treated with distinct combinations of dasatinib (*y*-axes) and trametinib (*x*-axes) ranging from 0 to 10 μmol/L. A heat map is overlaid to the proportions of viable cells remaining, colored according to the key provided from 1.0 (black, all cells) to 0 (red, no viable cells). **B,** Excess above BLISS matrices for ICR-B169 parental and trametinib-resistant clones treated with distinct combinations of dasatinib (*y*-axes) and trametinib (*x*-axes) ranging from 0 to 10 μmol/L. A heat map is overlaid to the excess score, colored according to the key provided from 0.4 (red, enhanced effects) to 0 (green, no difference). **C,** BLISS synergy maps for ICR-B169 parental and trametinib-resistant clones treated with distinct combinations of dasatinib (*y*-axes) and trametinib (*x*-axes) ranging from 0 to 10 μmol/L. The heat map represents the δ score colored according to the key provided from 30 (red, high degree of synergy) to −30 (green, antagonism).

Finally, we assessed whether this reciprocal sensitivity to targeted inhibition would lead to an effective combination therapy in MAPK-driven DIPG cells. We used the BLISS independence model to assess the combination of dasatinib and trametinib, which showed profound effects on cell viability in ICR-B169 parental cells, even at low doses of both drugs (Fig. 6A). This was in contrast to the trametinib-resistant clones, which unsurprisingly required high levels of MEK inhibitor to produce an effect (large black areas on heat map). This was more formally evaluated by calculating the excess above BLISS scores for each pairwise combination, identifying likely synergistic areas within the combinatorial matrix, highlighted in red on the heat map (Fig. 6B). A summary synergy score was calculated as the average excess response due to drug interactions above expectations, with the resulting value of 19.9 in the ICR-B169 parental cells indicating a high degree of formal synergy at the lowest doses of trametinib, an effect not seen in the resistant clones, in an analogous pattern to the cell viability score above (Fig. 6C). Notably, there was also a highly synergistic interaction of ulixertinib and trametinib in both parental and resistant clones, particularly with the T6 *MEK1*^K57N^ cells, which were largely resistant to ulixertinib as a single agent (Supplementary Fig. S7A–S7C).

**Figure 7.**
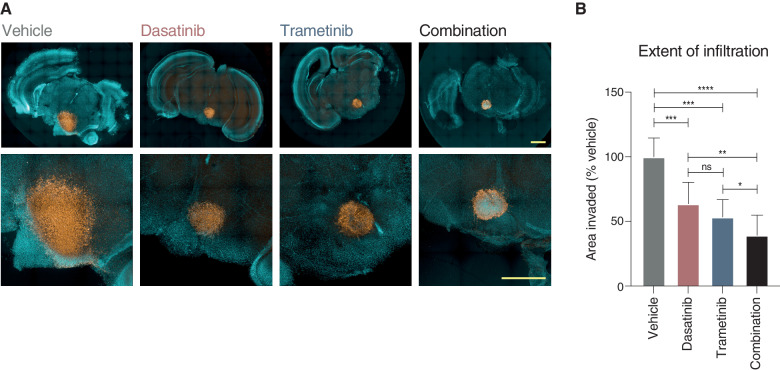
Efficacy of combined dasatinib and trametinib on *ex vivo* brain slice preparations. **A,** Coronal slices of normal mouse brain, counterstained with Hoechst 33342 (aqua), are implanted in the pontine region with ICR-B169 parental cells, stained with human nuclear antigen (orange), and treated for 4 days with 1 μmol/L dasatinib, 0.1234 μmol/L trametinib, or both compared with vehicle control. Scale bars, 2 mm. **B,** Bar plot of quantification of tumor cell infiltration across the brain parenchymal tissue as measured by the calculated area invaded compared with vehicle control. Plotted is the mean of at least six independent slices; error bars represent the SD. *, *P* < 0.05; **, *P* < 0.01; ***, *P* < 0.001; ****, *P* < 0.0001; ns, not significant, FDR-corrected *t* test.

To validate our proposed combinatorial approach, we established mouse *ex vivo* brain slice cultures, onto which we implanted ICR-B169 parental cells (Fig. 7A). Treatment with combined trametinib and dasatinib resulted in a significantly reduced growth capacity across the brain parenchymal tissue compared with vehicle (*P* = 0.0003, FDR-corrected *t* test) or either agent alone (*P* = 0.0057 vs. dasatinib; *P* = 0.050 vs. trametinib; FDR-corrected *t* test; Fig. 7B). Critically, there was a substantial reduction in cell number evident in the tumor cell core in the combination arm not observed with the single agents.

Thus, we present evidence for a subset of patients with DIPG to harbor MAPK pathway–activating mutations, as well as sensitivity to MEK inhibition *in vitro* with the rapid acquisition of consistent resistance mutations, and identify potentially efficacious combination approaches based on the common mechanisms underlying these events.

## Discussion

In the setting of a coclinical trial of prospectively established DIPG patient-derived models, we show the identification of biomarkers of response to trametinib, a selective reversible inhibitor of MEK1/2 that binds to the allosteric pocket of MEK. Specifically, these involve multiple nodes of the MAPK signaling pathway and include *PIK3R1*^N564D^, *NF1*^I1824S^, and *BRAF*^G469V^. These results suggest a possible rationale for the use of trametinib in DIPG, such as the ongoing phase II clinical trial called TRAM-01 (NCT03363217), which is exploring the use of trametinib in pediatric gliomas harboring MAPK alterations independently of the tumor entity (46).

*PIK3R1* ^N564D^ is an oncogenic hotspot mutation known to promote cell survival *in vitro* and oncogenesis *in vivo* (47) and lies within the regulatory subunit of PI3 kinase, resulting in loss of function, predicted to destabilize protein interaction, which may affect tumor suppressive function (FATHMM pathogenic score of 0.99; refs. 48, 49). Such *PIK3R1* oncogenic mutations have been shown to activate the MAPK pathway and exhibit sensitivity to MAPK inhibitors (50). *NF1*^I^*^1824^*^S^ lies in the neurofibromin chain and the lipid binding region, which may affect the protein folding and the tumor suppressor gene function (48); it has also been reported in a patient with neurofibromatosis type 1 (51) and is absent from population databases. The efficacy of MEK inhibitors has previously been shown in NF1-deficient glioblastoma cell lines (52), in addition to clinical benefit in refractory neurofibromatosis-associated glioma harboring the *NF1* mutation (53–55). By stochastic selection, an imbalance of VAF between 3-D and 2-D cultures from the same patient was observed in ICR-B181 (*PIK3R1*^N564D^) and ICR-B184 (*NF1*^I^*^1824^*^S^). Trametinib sensitivity was identified only in the mutant cultures, which also had higher basal levels of MAPK pathway activation, which would support the hypothesis that these mutations were responsible for the observed trametinib efficacy *in vitro.*

*BRAF* ^G469V^ is a class II *BRAF* mutation within the protein kinase domain, resulting in increased kinase activity and downstream MEK and ERK activation (3). *BRAF* class II mutations have constitutively activated BRAF dimers independent of RAS activation (3), and *BRAF*^G469V^ has been shown to confer sensitivity to trametinib in melanoma and lung cancer (4, 56). Despite this, in the context of DIPG, targeting this mutation with MEK inhibitor as a single agent *in vivo* and clinically failed to elicit a significant response. Although there may be issues with the treatment regimen used in our long-latency orthotopic xenografts, and the patient received trametinib only postprogression after several months on everolimus and radiation, there are also concerns over the emergence of resistance to MEK inhibition as previously described in melanoma or colorectal cancers (37–40). Such a process we showed to be active in DIPG cells through the acquisition in continuous exposure experiments *in vitro* of *MEK1*/*2* mutations, resulting in pathway reactivation and the irreversible resistance to trametinib. Although not observed in trametinib-insensitive CDX models *in vivo*, we did observe plausible variants that form part of a BRAF–MEK1/2 circuit, further underlying the importance of reactivation of this element of the signaling pathway in response to drug interventions.

MEK1 and MEK2 exhibit 85% peptide sequence homology (57). *MEK1*^K57N^ lies on the helix A domain within the N-terminal negative regulatory region and is associated with high levels of RAF-regulated activation of ERK signaling (31, 58). Interestingly, *MEK1*^K57N^ has been attributed to cause resistance to BRAF and MEK inhibitors *in vitro* and in patients with melanoma (45, 59). To the best of our knowledge, neither *MEK1*^I141S^ nor *MEK2*^I115N^ has been previously detected, although both mutations lie within the protein kinase domain affecting the allosteric pocket (45). Particularly, *MEK1*^I111N^, the equivalent of *MEK2*^I115N^, has been demonstrated to confer resistance *in vitro* to allosteric MEK inhibitors (31, 45). In our resistant clones, the presence of the *MEK1/2* mutations resulted in shift of 2 to 3 orders of magnitude of trametinib concentration required to inhibit MAPK signaling.

Notably, using integrated RNA-seq and phospho/total proteomics, a drift from a proneural to a mesenchymal phenotype was observed in the *MEK1/2*-mutant resistant clones, a feature also observed in patient-derived models inherently insensitive to trametinib. This has been broadly reported as a hallmark of metastasis and resistance to multitherapy in cancer (60, 61). In particular, glioma-initiating clones displaying drug resistance and radioresistance have been previously linked to a proneural–mesenchymal transition (62). In our models, this was also accompanied by a reciprocal sensitivity of the trametinib-resistant DIPG cells to the multikinase inhibitor dasatinib. In this context, phospho-kinase profiling highlighted Src-family kinases as the likely mediators of this response rather than PDGFR signaling. In other cancers, a proportion of *KRAS*-mutant cell lines resistant to trametinib was found to have a mesenchymal gene expression signature (63), and dasatinib has been reported to overcome mesenchymal transition–associated resistance to erlotinib in non–small cell lung cancers (64). In addition, a study has found that dasatinib sensitizes *KRAS*-mutant cancers to trametinib both *in vivo* and *in vitro* (65). In DIPG, the combined use of dasatinib and trametinib showed a high degree of synergy *in vitro* and on *ex vivo* brain slices and may represent a novel combinatorial approach in this disease.

It remains to be explored how best these combinations could be translated clinically in order to prevent or overcome resistance to MEK inhibitors. Critically, we observed consistent results in our primary patient-derived cultures, 2-D/3-D MAPK-altered isogenic cells, and the trametinib-resistant clones for the more clinically relevant brain-penetrant MEK inhibitors cobimetinib and binimetinib. Going forward, assessment of an intermittent multitherapy regimen to control population dynamics and potentially prevent emergence of treatment resistance to begin with is clearly warranted. In this context, mathematical modeling and adaptive drug design will be essential to determine treatment scheduling, exploiting the fitness trade-offs associated with resistance therapy (66).

## Methods

### Primary Patient-Derived Cell Cultures

DIPG biopsy tissue was shipped to our laboratory in Hibernate A transport media (ThermoFisher Scientific, A12475-01) at room temperature or minced with a sterile scalpel blade in DMEM/F12 (Life Technologies, 11320-074) supplemented with 0.2% BSA (Sigma-Aldrich, A1595) and 10% DMSO (Sigma-Aldrich, D2650), frozen at −80°C and sent on dry ice (Supplementary Table S1). Minced tissue was digested using Liberase DL. Supernatant was removed and the tissue/cells were resuspended in stem cell media and continuously pipetted to ensure dissociation before transfer to culture flasks to grow either attached on a laminin substrate (Merck Millipore, CC095) and/or in suspension as neurospheres in ultra-low attachment flasks (Sigma-Aldrich, CLS3815). The cells were grown at 37°C, 5% CO_2_ in stem cell media consisting of DMEM/F12 (Life Technologies, 11330-038), Neurobasal-A Medium (Life Technologies, 10888-022), HEPES Buffer Solution 1 mol/L (Life Technologies, 15630-080), MEM Sodium Pyruvate Solution 100 nmol/L (Life Technologies, 11360-070), MEM Non-Essential Amino Acids Solution 10 mmol/L (Life Technologies, 11140-050), and Glutamax-I Supplement (Life Technologies, 35050-061). The media were supplemented with B-27 Supplement (Life Technologies, 12587-010), 20 ng/mL recombinant Human-EGF (2B Scientific LTD, 100-26), 20 ng/mL recombinant Human-FGF (2B Scientific LTD, 100-146), 20 ng/mL recombinant Human-PDGF-AA (2B Scientific LTD, 100-16), 20 ng/mL recombinant Human-PDGF-BB (2B Scientific LTD, 100-18), and 2 μg/mL Heparin Solution (Stem Cell Technologies, 07980).

### Nucleic Acid Extraction

DNA and RNA were isolated from the same piece of tissue or cell pellet using ZR-Duet DNA/RNA Miniprep Plus (Zymo Research, D7001). If only DNA was extracted, the DNeasy Blood & Tissue Kit (Qiagen, 69581) was used, and if only RNA was extracted, the RNeasy Mini Kit protocol (Qiagen, 74104) was used. DNA and RNA quality were measured using a Nanodrop spectrophotometer (Thermo-Scientific). DNA concentration was determined using a Qubit fluorometer (Thermo Fisher Scientific, Q32850 and Q32851). RNA integrity was analyzed and quantified using a 4200-Tapestation (Agilent).

### Whole-Exome Sequencing

Libraries were prepared from 50 to 200 ng DNA using the Kapa HyperPlus Kit, and DNA was indexed using 8-bp TruSeq-Custom Unique Dual Index Adapters (IDT). Libraries were pooled in 8-plex (250–500 ng of each library) by equal mass and normalized to the lowest mass sample. Samples were then hybridized overnight (O.N.; 16–18 hours) with the xGen Exome Research panel v1 (IDT) and sequenced on an Illumina NovaSeq 6000 system using the S2-200 Reagent Kit (Illumina, 20012861) or the SP Reagent Kit (Illumina, 20027465). Capture reads were aligned to the GRCh37/Hg19 build of the human genome using bwa bwa v0.7.12 (bio-bwa.sourceforge.net), PCR duplicates were removed with PicardTools 1.94 (pcard.sourceforge.net), and BEDTools was used for quality control and generation of metrics for each sample. Single-nucleotide variants were called using the Genome Analysis Tool Kit v3.4-46 based on current best practices using the Unified Genotyper (broadinstitute.org/gatk/). Variants were annotated using the Ensembl Variant Effect Predictor v74 (ensembl.org/info/docs/variation/vep) incorporating SIFT (sift.jcvi.org) and PolyPhen (genetics.bwh.harvard.edu/pph2) predictions, COSMIC v64 (sanger.ac.uk/genetics/CGP/cosmic/), dbSNP build 137 (ncbi.nlm.nih.gov/sites/SNP), ExAc, and ANNOVAR annotations. Copy number was obtained by calculating log_2_ ratios of tumor/normal coverage binned into exons of known Ensembl genes, smoothed using circular binary segmentation (DNAcopy, www.bioconductor.org), and processed using in-house scripts in R. Interaction networks were analyzed and visualized by STRING (string-db.org).

### RNA-seq

At least 150 ng RNA was sequenced at Eurofins Genomics. Strand-specific cDNA libraries were made by purification of poly-A containing mRNA molecules followed by mRNA fragmentation and random primed cDNA synthesis (strand-specific). Adapter-ligation and adapter-specific PCR amplification was performed before sequencing on Illumina sequencers (HiSeq or NovaSeq) using 150-bp paired-end reads chemistry according to the manufacturer's instructions. RNA-seq data were aligned with STAR and summarized as gene-level fragments per kilobase per million reads sequenced using BEDTools and HTSeq. Following rlog transformation and normalization, differential expression was assigned with DESeq.2. Fusion transcripts were detected using chimerscan version 0.4.5a filtered to remove common false positives. GSEA was carried out using the R package fastGSEA (fGSEA) based on curated canonical pathways (MsigDB, Broad Institute).

### Methylation

A total of 50 to 500 ng DNA was bisulfite modified using the EZ DNA Methylation-Direct Kit (Zymo, D5006) and loaded onto the Illumina Infinium MethylationEPIC BeadChip, and the array intensities were read on the Illumina iScan system at the University College London Genomics Centre according to the manufacturer's instructions. Methylation data from the Illumina Infinium HumanMethylation850 BeadChip were preprocessed using the minfi package in R (v11b4). DNA copy number was recovered from combined intensities using the conumee package. The Heidelberg brain tumor classifier (ref. 67; molecularneuropathology.org) was used to assign a calibrated score to each case, associating it with one of the 91 tumor entities that feature within the current classifier. Clustering of β values from methylation arrays was performed based on correlation distance using a Ward algorithm. DNA copy number was derived from combined log_2_ intensity data based on an internal median processed using the R packages minfi and conumee to call copy number in 15,431 bins across the genome.

### ddPCR

Custom TaqMan-based quantitative PCR genotyping assays (Applied Biosystem, Thermo Scientific) were designed to specifically detect *PIK3R1*^N564D^ and *MEK1*^K57N^, *MEK1*^I141S^, and *MEK2*^I115N^ mutations (Supplementary Table S2). ddPCR was performed by using the Bio-Rad automated droplet generator (BioRad, QX200 AutoDG) and the Bio-Rad QX200 droplet reader, based on the manufacturer's guidelines. Results were analyzed using Bio-Rad QuantaSoft Analysis Pro software. At least two single FAM-positive droplets were required to call a sample positive.

### Drug Assays

Primary cells were incubated in 96- or 384-well plates for 3 days before adding the compounds with serial dilution at 10 different concentrations, and 8 days later (endpoint, day 11) cell viability was measured using CellTiter-Glo (2.0, or 3-D assays as appropriate; Promega). Relative luminescence units (RLU) for each well were normalized to the median RLU from the DMSO control wells as 100% viability. At least two duplicates per drug condition were performed as well as a minimum of three independent biological replicates. GI_50_ values (drug concentration causing 50% inhibition of cell proliferation) were calculated using GraphPad Prism, and the curves show the mean ± SE of the replicates per condition measured. All compounds were purchased from Selleckchem except for CUDC-907 and PTC-209, which were obtained from Apexbio. The compounds were diluted in DMSO to a final concentration of 10 mmol/L. Drug plates were prepared using the acoustic liquid handler Echo 550 (Labcyte). Each plate included six compounds at eight different concentrations as well as a cytotoxic chemotherapeutic (campto-thecin) as a positive control, as well as DMSO as a negative control. Drug combinations were assessed by adding one compound in rows and another in columns with serial dilutions resulting in a 6 × 10 dose matrix using different fold dilutions manually prepared. The stand-alone web application SynergyFinder (https://synergyfinder.fimm.fi) was used for interactive analysis and visualization of multidrug combination profiling data following the BLISS independence model (68, 69).

### Generation of Resistant Clones

ICR-B169 resistant clones were established by culturing the parental line in escalating concentrations of trametinib from 0.05 μmol/L (GI_50_) to 1 μmol/L in an exponential stepwise manner or by exposing the cells to a constant concentration of trametinib of 0.5 μmol/L (GI_80_ value). Both methods were performed using two technical replicates and two independent biological replicates for a total of eight derived cultures plus four replicate control flasks that were treated with the same concentration of DMSO in parallel to the establishment of the resistant cells. Cells were maintained in DMSO for only two passages to keep the baseline clonal population as close as possible to the parental culture. A total of 1 to 1.4 × 10^6^ cells were seeded in a T75 flask for the resistance assay, and 2 to 3 days later the media with trametinib were added at the appropriate concentration or DMSO to the control flasks. For the escalating-dose approach, 10 different concentrations were used with an exponential increment, and the same dose was added a total of six times. During the generation of resistance, the stem cell media containing the drug or DMSO were replaced three times a week and the cells split when they reached 90% confluency.

### Capillary-Based Protein Quantification

Cells were scraped from the flask and collected in media or cold PBS and then centrifuged at 1,300 rpm for 5 minutes, resuspended in 1 mL cold PBS, and centrifuged at 4,000 rpm for 4 minutes. The supernatant was then removed and the pellet was resuspended in 50 to 100 μL cold lysis buffer (Cell Signaling Technologies, 9803) containing protease inhibitor cocktail mini-tablet (Roche, Diagnostics, 11836153001) and phosphatase inhibitors (Sigma-Aldrich, P044 and P5726). Lysates were sonicated for 10 seconds at 40% amplitude, spun at 14,000 rpm for 10 minutes in a cold microfuge, and quantified using the BCA Protein Assay Kit (Thermo Fisher, 23225). Capillary electrophoresis was conducted using the automated Wes system (ProteinSimple) with the 12- to 230-kDa Separation module (SM-W004) and the anti-rabbit detection module (DM-001) following the manufacturer's instructions, and analyzed with Compass software. The following primary antibodies were used: AKT (1:50, Cell Signaling Technologies, 9272), phospho-AKT^Ser473^ (1:50, Cell Signaling Technologies, 4060), ERK1/2 (1:100, Cell Signaling Technologies, 9102), pERK^T202/Y204^ 1:100 (Cell Sig-naling Technologies, 9101), MEK1/2 (1:50, Cell Signaling Technologies, 9122), pMEK1/2^S217/221^ (1:50, Cell Signaling Technologies, 9121), α-Actin (1:200, Cell Signaling Technologies, 6487), and the AKT/MAPK pathway antibody cocktail (1:25, Abcam, 151279). Goat Anti-Rabbit HRP conjugate (ProteinSimple, 042-206) was used as a secondary antibody.

### Phospho-Kinase Profiling

Capillary electrophoresis was conducted using the automated Wes system (ProteinSimple) with the 12- to 230-kDa Separation module (SM-W004) and the anti-rabbit detection module (DM-001) following the manufacturer's instructions and analyzed with Compass software. In each run, each well contained an antibody of interest (9101L, pERK1/2^T202/Y204^; 9102L, total ERK1/2; Cell Signaling) and normalized to loading control antibody (6487S, α-actinin; Cell Sig-naling) in each well.

Human Phospho-Kinase Arrays (R&D Systems, ARY003C) were placed in 1.5 mL array buffer 1 and agitated gently for 1 hour at room temperature. Array buffer was substituted by dilute lysate (100 μg/mL) and left O.N. at 4°C. Membranes were subsequently washed three times in wash buffer for 10 minutes and then incubated in detection antibody for 2 hours at room temperature. The arrays were rinsed a further three times in wash buffer and then covered by Streptavidin-HRP in 1× Array Buffer (Thermo Fisher) and incubated at room temperature for 30 minutes. They were visualized using the LI-COR Imaging System and analyzed using the Quick Spots Image Analysis Software (R&D Systems). Briefly, the pixel density of each spot on the array was measured, and then the average signal of the pair of duplicate spots representing each pRTK was calculated. Finally, the averaged background signal from each RTK spot was subtracted.

For treatment with trametinib or dasatinib, cells were incubated in complete media with vehicle (DMSO) or increasing concentrations of drug (0.01, 0.1, 1, and 10  μmol/L), and protein was collected at 30 minutes posttreatment. Samples were lysed by using lysis buffer (CST) containing phosphatase inhibitor cocktail (Sigma-Aldrich) and protease inhibitor cocktail (Roche Diagnostics).

### Proteomics

Cell pellets were lysed in 5% SDS/100 mmol/L triethylammonium bicarbonate buffer with probe sonication and heating at 95°C for 10 minutes, and protein concentration was measured by the Pierce 660-nm Protein Assay. Then, 300 μg protein was reduced with 5 mmol/L Tris (2-carboxy-ethyl)-phosphin-HCl, alkylated by iodoacetamide, and then purified by methanol/chloroform precipitation. Trypsin was added at a 1:30 ratio (trypsin/proteins) for 18 hours of digestion at 37°C. Next, 150 μg of peptides per sample was tandem mass tag (TMT) labeled as instructed by the manufacturer (Thermo Scientific). The TMT-labeled peptide mixture was fractionated by C18 column at pH 10, and fractions were collected and pooled. Phospho-peptide enrichment used the High-Select Fe-NTA Phospho-peptide Enrichment Kit. Samples were analyzed on an Orbitrap Fusion Lumos coupled with an Ultimate 3000 RSLCnano System (Thermo Scientific) using the Synchronous Precursor Selection method with dynamic exclusion enabled (as reported in PMID 28854368). All raw files were processed in Proteome Discoverer 2.3 (phospho-proteome) or 2.4 (full proteome; Thermo Fisher) using the SequestHT search engine. Spectra were searched against reviewed Uniprot *Homo sapiens* entries (November 2019) and an in-house contaminant database. Search parameters for phospho-proteome were trypsin with up to 2 miscleavage sites; mass tolerances at 20 ppm for Precursor and 0.02 Da for fragment ions; dynamic modifications of deamidated (N, Q), oxidation (M), and phospho (S, T, Y); and static modifications of carbamidomethyl and TMT6plex (peptide N-terminus and K). For full proteome, fragment ion tolerance was 0.5 Da, with dynamic modification of oxidation (M), acetylation (Protein N-terminus), and static modifications of carbamidomethyl and TMT6plex (peptide N-terminus and K). Peptides were validated by Percolator with *q* value set at 0.05. Phosphorylation site localization probabilities were computed by the ptmRS node. The TMT10plex reporter ion quantifier included 20-ppm integration tolerance on the most confident centroid peak at the MS3 level of unique peptides. Peptides with average reported S/*N* > 3 were used for protein quantification, and the SPS mass matches threshold was set at 55%. Only master proteins were reported.

### Orthotopic Xenograft Studies

All *in vivo* experiments were approved by the local Animal Welfare and Ethics Review Board at the Institute of Cancer Research and carried out in accordance with the UK Home Office Animals (Scientific Procedures) Act of 1986, the UK National Cancer Research Institute guidelines for the welfare of animals in cancer research, and the ARRIVE (Animal Research: Reporting *In Vivo* Experiments) guidelines (70). A single cell suspension was prepared from cells from tissue [patient-derived xenograft (PDX), *n* = 9] immediately prior to implantation in NOD.Cg-*Prkdc^scid^ Il2rg^tm1WjI^*/SzJ (NSG) mice (Charles River). Animals were anesthetized with 4% isoflurane and maintained at 2% to 3% isoflurane delivered in oxygen (1 L/min). Mice were placed on a stereotactic apparatus for orthotopic implantation, with coordinates x = +1.0, z = −0.8, y = −4 mm from the lambda used for delivery to the pons. Then, 5 μL of cell suspension (250,000 cells) was stereotactically implanted per animal, using a 25-gauge SGE standard fixed needle syringe (SGE 005000) at a rate of rate of 1 μL/min for PDX and 2 μL/min for CDX using a digital pump (HA1100, Pico Plus Elite; Harvard Apparatus). For the trametinib efficacy study, mice (29–33 days old) were randomized according to tumor volume determined by MRI into two groups: group 1 included vehicle [10% DMSO, w/v hydroxyproylbetacyclodextrin dissolved in PBS, oral gavage (PO), every day], and group 2 included trametinib (1 mg/kg, PO, every day). Animals were treated for 8 weeks, 5 days on, 2 days off, and treatment started at day 55 postinjection. ^1^H MRI was performed using a horizontal bore Bruker Biospec 70/20 equipped with physiologic monitoring equipment (SA Instruments) using a 2-cm × 2-cm mouse brain array coil. Anesthesia was induced using 3% isoflurane delivered in oxygen (0.5 L/min) and maintained at 1% to 2%. Core body temperature was maintained using a thermoregulated water-heated blanket. Following optimization of the magnetic field homogeneity using a localized map shim over the whole brain, a rapid acquisition with relaxation enhancement (RARE) T_2_-weighted sequence (repetition time = 4,500 ms, effective echo time = 36 ms, two averages, RARE factor = 8, in-plane resolution 98 μm × 98 μm, 1-mm-thick contiguous slices) was used for localization and assessment of tumors. Mice were weighed twice a week and sacrificed by cervical dislocation upon deterioration of condition, and tissue was taken for further analysis. For immunohistochemistry, sodium citrate (pH 6.0) heat-mediated antigen retrieval was performed and staining was carried out using antibodies directed against human nuclear antigen (1:100, Millipore, MAB4383) diluted into 1% Tris buffer solution with 0.05% Tween-20 and incubated 1 hour at room temperature. Novocastra Novolink Polymer Detection Systems Kit (Leica Biosystem, RE-7150) was used for detection. Slides were mounted using a Leica CV Ultra mounting medium (Leica, 070937891).

### Coronal Whole-Brain Organotypic Slice

All animal procedures were under the European Communities Council Directive N. 2010/63/EU and the Italian Ministry of Health guidelines (DL 26/2014) and approved by the Italian Ministry of Health and by the local Institutional Animal Care and Use Committee at Istituto Superiore di Sanità (Rome, Italy; protocol n. D9997.N.BYG, 2019). Coronal whole-brain organotypic slices encompassing the pons were prepared from CD1 mice pups (postnatal days 6–7; Charles River) as previously described (71), with some modifications. In brief, mice were decapitated and brains rapidly dissected and placed in ice-cold artificial cerebrospinal fluid containing (in mmol/L): 126 NaCl, 3.5 KCl, 1.2 NaH_2_PO_4_, 1.2 MgCl_2_, 2 CaCl_2_, 25 NaHCO_3_, and 11 glucose (pH 7.3), saturated with 95% O_2_ and 5% CO_2_. The brain was then embedded in 3% SeaPlaque agarose (Lonza) in PBS and 300-μm-thick coronal slices were cut on a vibrating microtome (Campden Instruments), constantly cooled, and oxygenated. Each slice was transferred onto a porous membrane (0.45-μm pore size; Millipore), placed on a Millipore culture insert, and inserted into 6-well plates with 1.2 mL of cell culture medium/well, where the inserts were placed. The slices were incubated at 35°C, 5% CO_2_ for 7 days before the experiments to allow the inflammatory reaction following the mechanical procedure to subside. Following the first day of culture, the medium was replaced with fresh medium and, from that time, changed every 48 hours. Seven days after slices were sectioned, ICR-B169 parental neurospheres with a diameter of 250 to 300 μm (1 neurosphere/slice) were implanted on the pontine area, and following 3 days of coculture, preparations were treated for 4 days with 1 μmol/L dasatinib, 0.1234 μmol/L trametinib, or both compared with vehicle control (DMSO). After this time, slices were fixed with 10% buffered formalin for 2 hours at room temperature (RT) and washed twice with PBS for 10 minutes. Slices were then permeabilized with 1% Triton in PBS for 90 minutes and then blocked with 10% goat serum + 1% BSA + 0.1% Triton for 60 minutes. Slices were incubated O.N. with anti-human nuclei antibody (Millipore, 1:300). Slices were washed with PBS twice for 10 minutes and incubated with the secondary antibody (Alexa Fluor-555 goat anti-mouse, 1:500 O.N.; Invitrogen). Hoechst33342 was used as a counterstain (1:10,000 in PBS for 45 minutes at RT; Invitrogen). Images were taken on an Operetta CLS (PerkinElmer) in confocal mode (10×; z-stack 42 μm).

### Statistical Analysis

Statistical analysis was carried out using R 3.3.0 (THe R Project for Statistical Computing) and GraphPad Prism 8 (GraphPad Software).

Categorical analyses were carried out by ANOVA, with multiple comparisons subjected to the Dunnett test. *In vitro* dose-response effects were compared by AUC analysis. Normalized and scaled abundances proteins and phospho-peptides were compared by using a Student *t* test. For these and gene expression data analysis, multiple testing corrections were made using FDR according to Benjamini and Hochberg. Effects of drug treatment on survival as the primary endpoint and overall survival in the orthotopic *in vivo* models were assessed using the Mantel Cox log-rank test. Adjusted *P* values <0.05 were considered significant.

### Data Accessibility

All newly generated sequencing data have been deposited in the European Genome-phenome Archive (ebi.ac.uk/ega) with accession number EGAS00001004495 (sequencing) or ArrayExpress (ebi.ac.uk/arrayexpress/) with accession number E-MTAB-9282 (methylation arrays). Proteomic data have been deposited in the Proteomics Identifications Database (PRIDE; ebi.ac.uk/pride) with accession number PXD019701. Curated gene-level mutation, copy number, and expression data are provided as part of the pediatric-specific implementation of the cBioPortal genomic data visualization portal (pedcbioportal.org).

## Supplementary Material

Supplementary Data

Supplementary Data

Supplementary Data

Supplementary Data

Supplementary Data
