# Supplementary material for: DIPG Harbors Alterations Targetable by MEK Inhibitors, with Acquired Resistance Mechanisms Overcome by Combinatorial Inhibition
Source: Cancer Discov. 2022 Mar 8;12(3):712–29. doi: 10.1158/2159-8290.CD-20-0930 (PMC7612484; doi:10.1158/2159-8290.CD-20-0930)
Supplement: Supplementary Data [file cd-20-0930_supp.pdf]

SUPPLEMENTARY FIGURES

Supplementary Table S1 – *Molecular characterisation of patient-derived primary DIPG cells established as part of a prospective co-clinical trial*. (A) Short tandem repeat profiling of biopsy samples (-TUM), blood (-NORM) and *in vitro* cultures grown in (-2D) and (-3D). (B) Somatic mutations, amplifications (AMP) and homozygous deletions (HOMDEL) in all profiled samples. (C) MNP methylation classifier scores for all profiled samples. (D) Area under curve (AUC) values for all screened cells.

Supplementary Table S2 – *ddPCR primer sequences.* Sequences provided for custom assays to detect *PIK3R1*_N564D, *MEK1*_K57N, *MEK1*_I141S and *MEK2*_I115N.

Supplementary Table S3 – *Gene set enrichment analysis.* Top-scoring GSEA hits from differential expression analysis of RNAseq data of (A) trametinib-resistant ICR-B169-clones compared to parental, and (B) trametinib sensitive compared to insensitive patient-derived models from our BIOMEDE panel. Top 50 positively- and negatively enriched signatures are provided, with those selected for figures highlighted.

Supplementary Table S4 – *Phospho-kinase array profiling*. Raw (averaged) and normalised counts for individual probes on two phospho-kinase arrays, hybridised with protein from ICR-B169 T6 cells treated with increasing concentrations of dasatinib *in vitro*.

Supplementary Figure S1

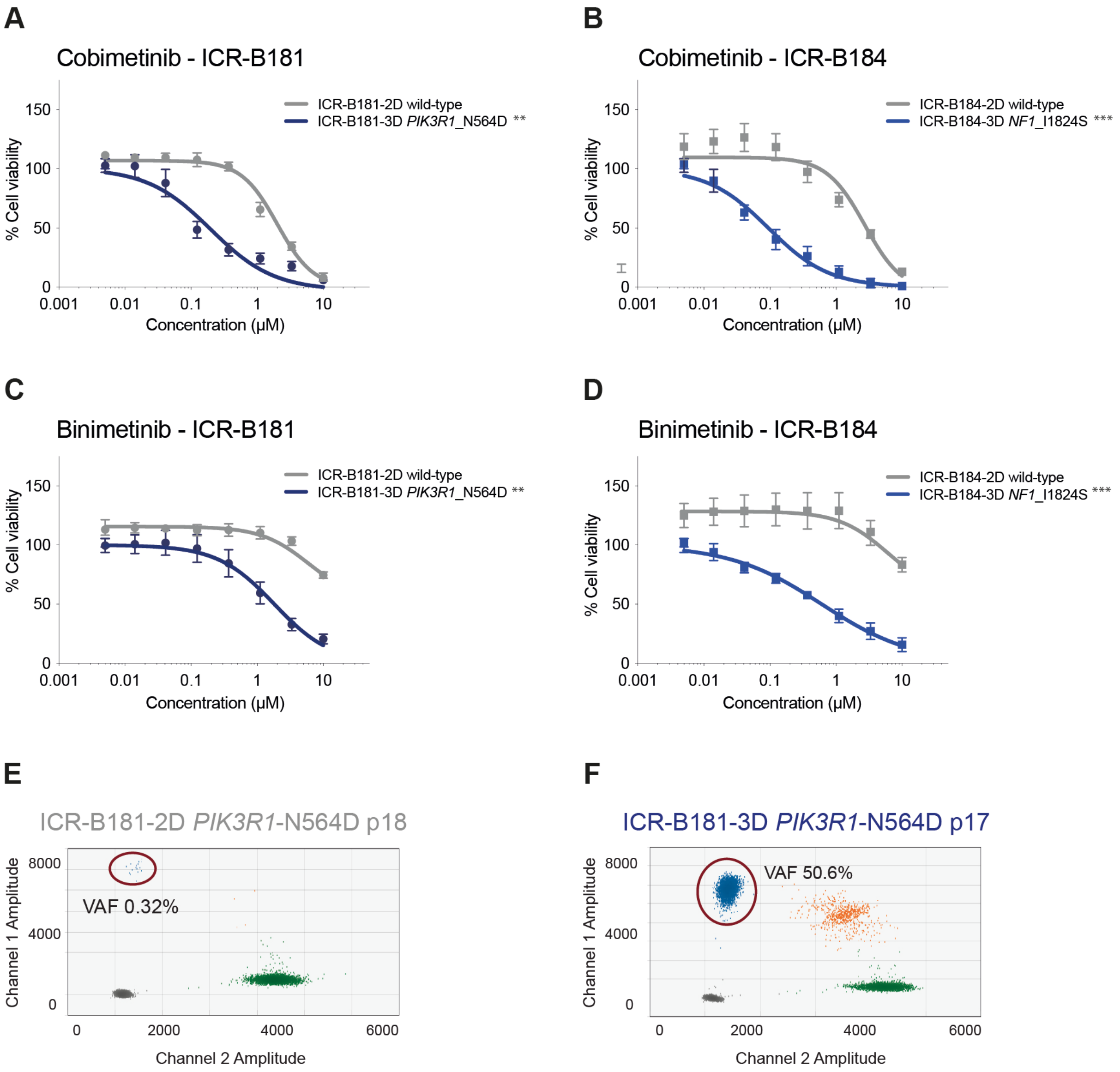

Supplementary Figure S1 – *Cross-sensitivity of MAPK-altered pairs of patient-derived DIPG cells to MEK inhibitors in vitro.* (A) Dose-response validation curves for cobimetinib tested against ICR-B181 cells *in vitro* grown in 3D (*PIK3R1*_N564D, blue) and 2D (*PIK3R1* wild-type, grey). (B) Dose-response curves for cobimetinib tested against ICR-B184 cells *in vitro* grown in 3D (*NF1*_I1824S, blue) and 2D (*NF1* wild-type, grey). (C) Dose-response validation curves for binimetinib tested against ICR-B181 cells *in vitro* grown in 3D (*PIK3R1*_N564D, blue) and 2D (*PIK3R1* wild-type, grey). (D) Dose-response curves for binimetinib tested against ICR-B184 cells *in vitro* grown in 3D (*NF1*_I1824S, blue) and 2D (*NF1* wild-type, grey). Concentration of compound is plotted on a log scale (x axis) against cell viability (y axis). Mean

plus standard error are plotted from at least n=3 experiments. ***p<0.001, **p<0.01, AUC t-test. (E) ddPCR amplitude plot for *PIK3R1* wild-type (x axis) and N564D mutation (y axis) for ICR-B181-2D cells, with mutant droplets highlighted and coloured in blue. (F) ddPCR amplitude plot for *PIK3R1* wild-type (x axis) and N564D mutation (y axis) for ICR-B181-3D cells, with mutant droplets highlighted and coloured in blue. *PIK3R1* wild-type droplets are shown in green, double positive droplets are shown in orange and empty droplets with no DNA are shown in grey.

Supplementary Figure S2

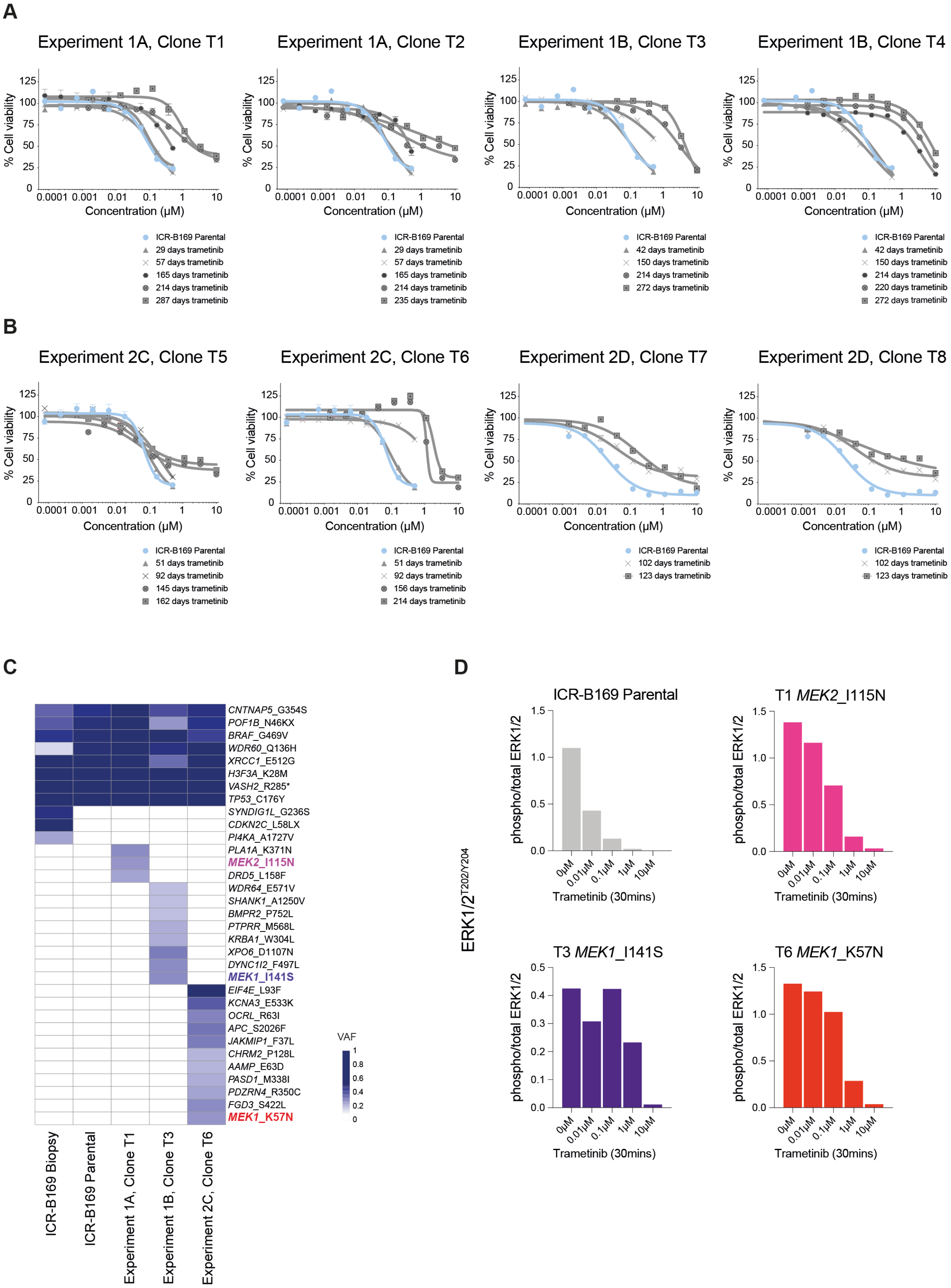

Supplementary Figure S2 – *Emergence of trametinib-resistant DIPG cells in continuous exposure experiments.* (A) Dose-response curves for trametinib tested against ICR-B169 parental cells (light blue) and cells grown under continuous exposure to inhibitor at different time points (shades of grey), taking Approach 1 consisting of exponential increase of trametinib over time. Concentration of compound is plotted on a log scale (x axis) against cell viability (y axis). Mean plus standard error are plotted from at least n=3 experiments. (B) Dose-response curves for trametinib tested against ICR-B169 parental cells (light blue) and cells grown under continuous exposure to inhibitor at different time points (shades of grey), taking Approach 2 consisting of a constant concentration of trametinib at IC_{80}. Concentration of compound is plotted on a log scale (x axis) against cell viability (y axis). Mean plus standard error are plotted from at least n=3 experiments. (C) Exome sequencing of ICR-B169 parental and subclones 1A.T1, 1B.T3 and 2C.T6 from continuous exposure experiments. Individual mutations are coloured according to their variant allele frequency. Mutations in *MEK1* and *MEK2* are coloured, and in bold. (D) Barplot of quantitative capillary phospho-protein assessment of phospho-ERK1/2$^{T202/Y204}$, plotted as a ratio to total ERK1/2, for ICR-B169 parental cells and trametinib-resistant clones, treated with increasing concentrations of trametinib for 30 mins.

Supplementary Figure S3

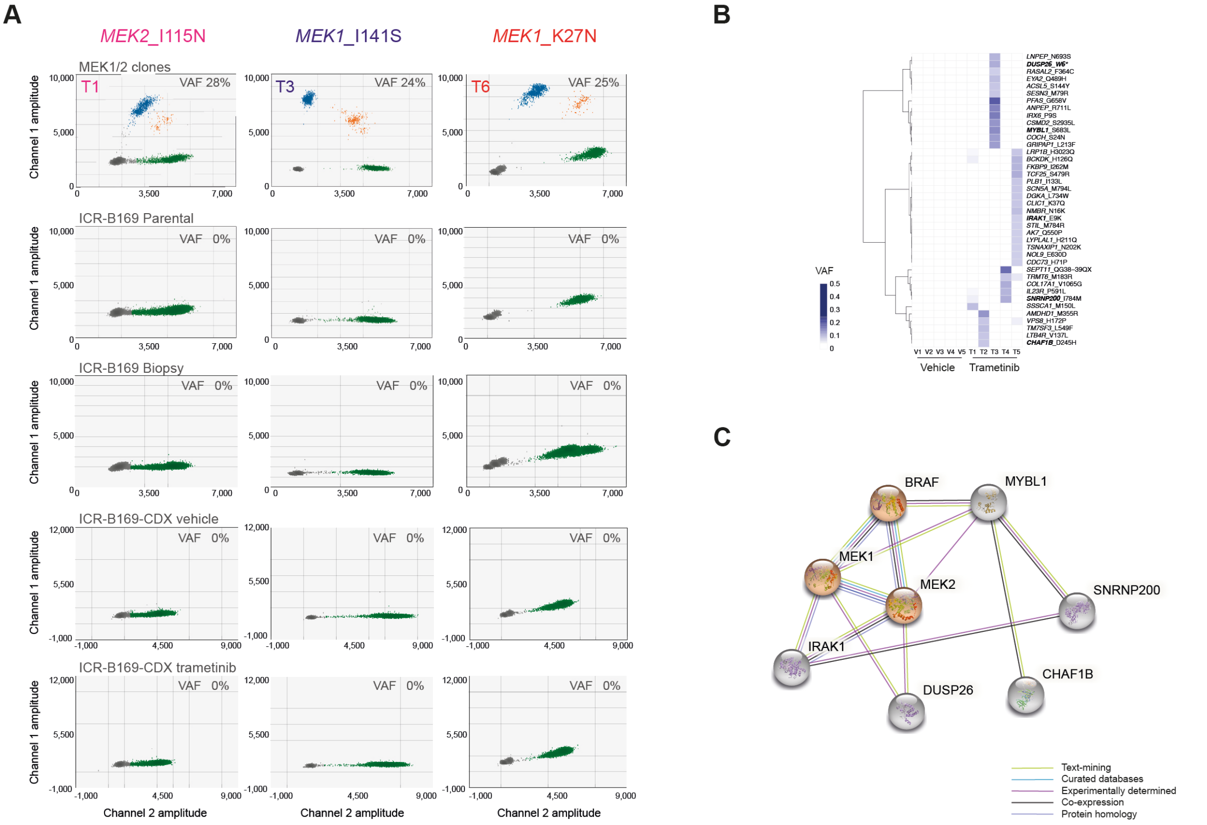

Supplementary Figure S3 – *Assessment of acquired mutations in trametinib-resistant DIPG models.* (A) ddPCR amplitude plot for *MEK2*_I115N (pink), *MEK1*_I141S (purple) and *MEK1*_K57N (red) in the respective positive trametinib-resistant subclones, ICR-B169 parental cells, ICR-B169 biopsy tissue, ICR-B169-CDX tumours treated with vehicle, and ICR-B169-CDX tumours treated with trametinib. *MEK1/2* wild-type (x axis) and relevant mutations (y axis) are plotted for each, with mutant droplets highlighted and coloured in blue. *MEK1/2* wild-type droplets are shown in green, double positive droplets are shown in orange and empty droplets with no DNA are shown in grey. (B) Exome sequencing of ICR-B169 parental CDX samples treated with trametinib. Individual mutations are coloured according to their variant allele frequency. Mutations with interactions within one degree of BRAF, MEK1 and MEK2 are highlighted in bold. (C) STRING protein-protein interaction network for genes in which variants were identified in exome sequencing of trametinib-treated ICR-B169 CDX xenografts. Interactions are coloured according to the evidence on which they are based.

Supplementary Figure S4

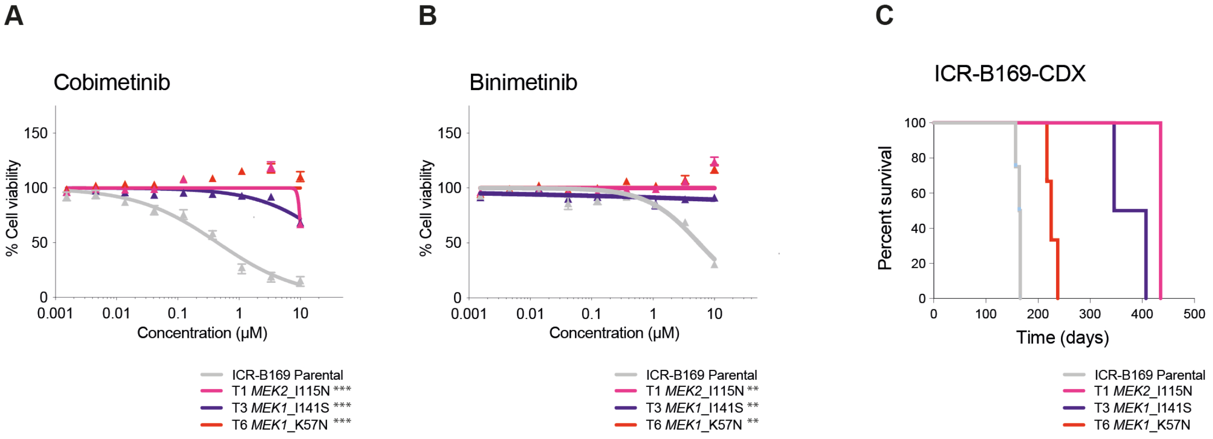

Supplementary Figure S4 – *Cross-resistance of trametinib-resistant ICR-B169 clones to MEK inhibitors in vitro.* (A) Dose-response validation curves for cobimetinib tested against ICR-B169 parental and trametinib-resistant clones *in vitro*. (B) Dose-response curves for binimetinib tested against ICR-B169 parental and trametinib-resistant clones *in vitro*. Parental, grey; T1 *MEK2*_I115N, pink; T3 *MEK1*_I141S, purple; T6, *MEK1*_K57N, red. Concentration of compound is plotted on a log scale (x axis) against cell viability (y axis). Mean plus standard error are plotted from at least n=3 experiments. ***p<0.001, **p<0.01, AUC t-test. (C) Survival curves for mice bearing ICR-B169 cell-derived orthotopic xenografts, parental and trametinib-resistant clones. Parental, grey; T1 *MEK2*_I115N, pink; T3 *MEK1*_I141S, purple; T6, *MEK1*_K57N, red.

Supplementary Figure S5

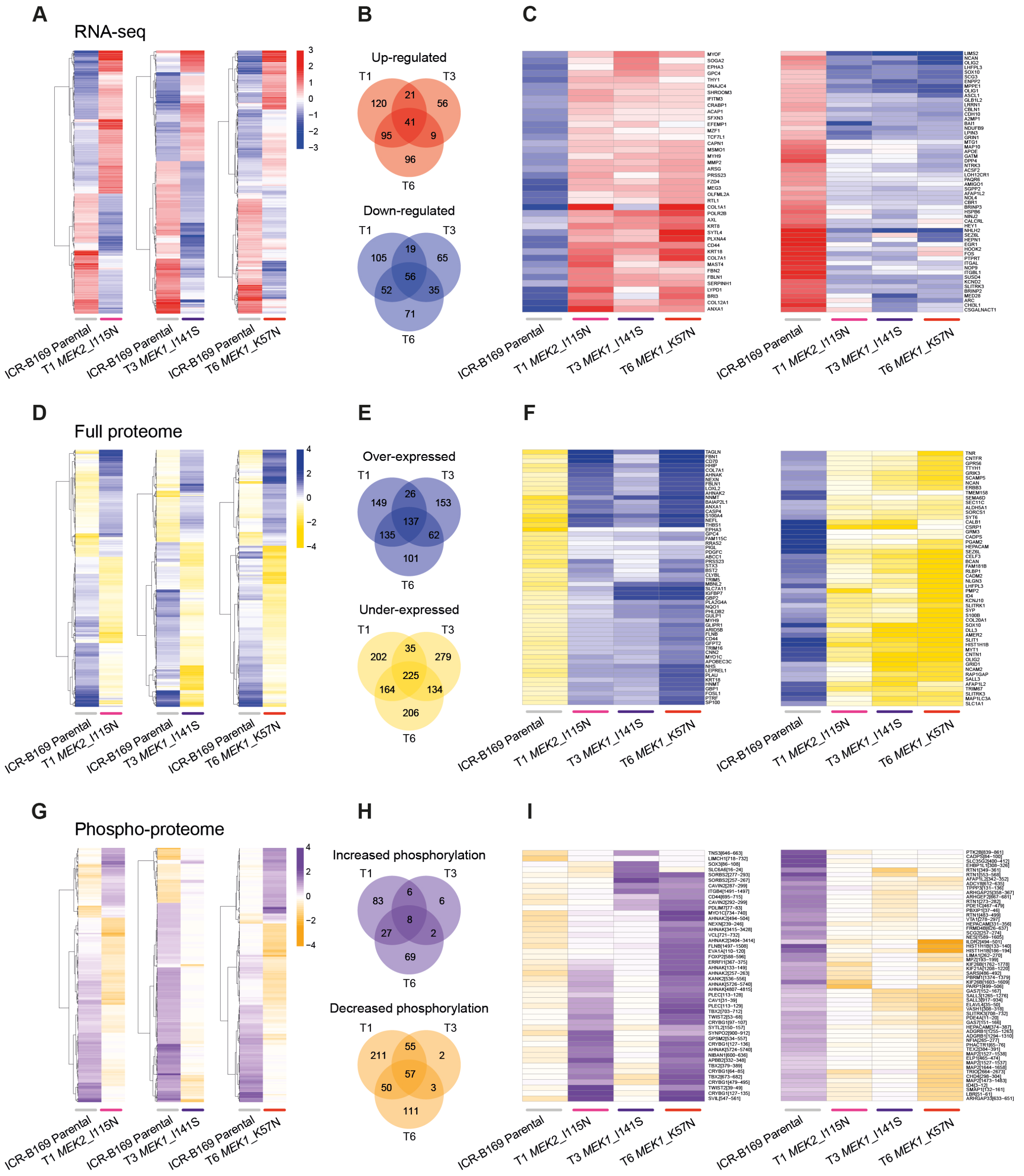

Supplementary Figure S5 – *Comprehensive gene and protein expression profiling of trametinib-resistant DIPG cells.* (A) Differentially expressed genes in individual trametinib-resistant clones compared to ICR-B169 parental, clustered by rows, and coloured according to the keys provided. (B) Venn diagrams showing overlap of differentially up- and down-regulated genes in the three resistant clones. (C) Heatmap of common up- and down-

regulated genes in the resistant clones compared to ICR-B169 parental. T1 *MEK2*_I115N, pink; T3 *MEK1*_I141S, purple; T6, *MEK1*_K57N, red. (D) Differentially expressed proteins in individual trametinib-resistant clones compared to ICR-B169 parental, clustered by rows, and coloured according to the keys provided. (E) Venn diagrams showing overlap of differentially over- and under-expressed proteins in the three resistant clones. (F) Heatmap of common over- and under-expressed proteins in the resistant clones compared to ICR-B169 parental. T1 *MEK2*_I115N, pink; T3 *MEK1*_I141S, purple; T6, *MEK1*_K57N, red. (G) Differentially phosphorylated sites in individual trametinib-resistant clones compared to ICR-B169 parental, clustered by rows, and coloured according to the keys provided. (H) Venn diagrams showing overlap of sites with differential increased and decreased phosphorylation in the three resistant clones. (I) Heatmap of common increased and decreased phosphorylation in the resistant clones compared to ICR-B169 parental. T1 *MEK2*_I115N, pink; T3 *MEK1*_I141S, purple; T6, *MEK1*_K57N, red.

Supplementary Figure S6

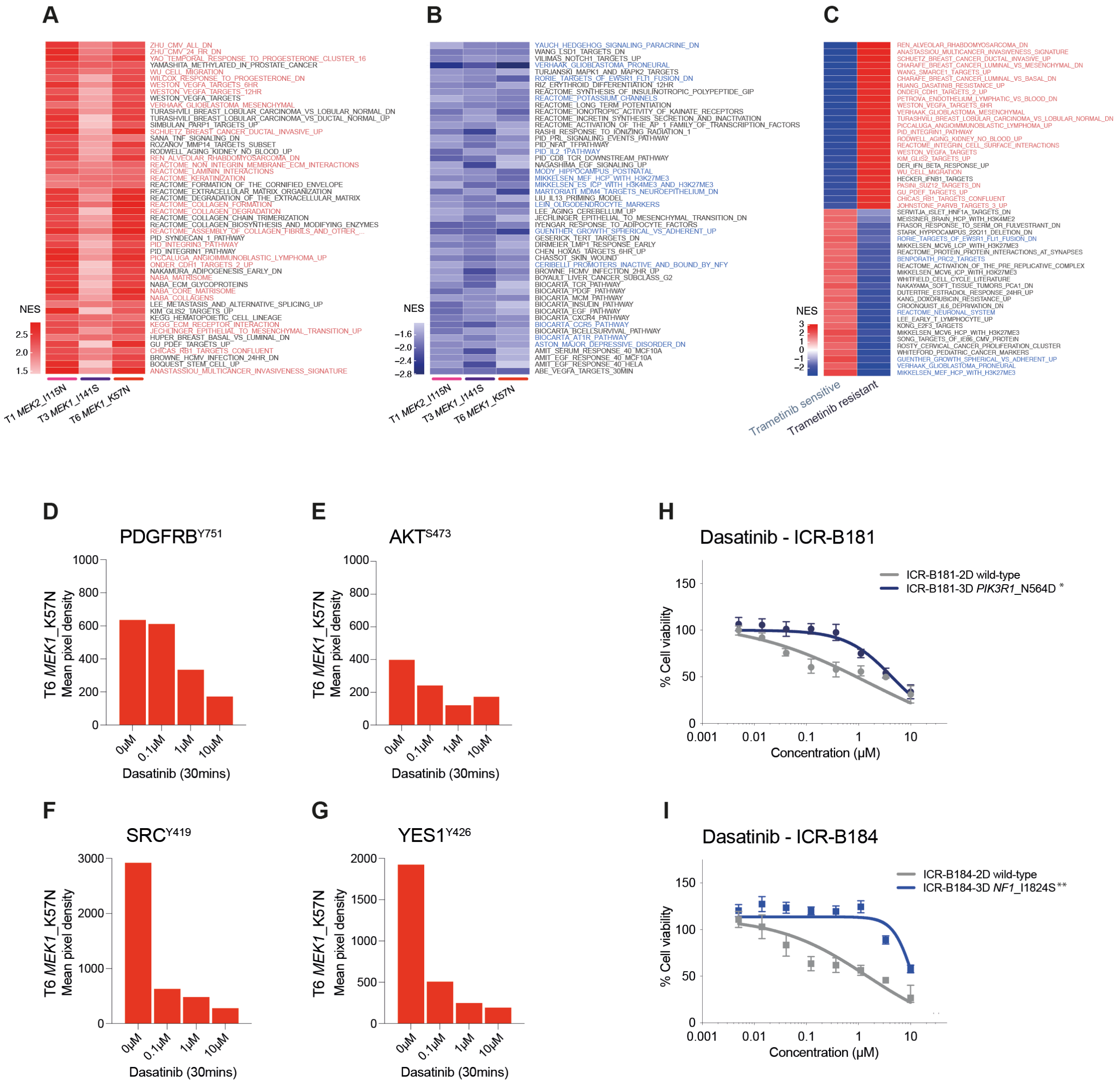

Supplementary Figure S6 – *Pathway enrichment in trametinib-resistant clones and inherently insensitive primary patient-derived cells.* (A) Significantly enriched up-regulated gene expression signatures in trametinib-resistant clones compared to ICR-B169 parental cells. (B) Significantly enriched down-regulated gene expression signatures in trametinib-resistant clones compared to ICR-B169 parental cells. (C) Significantly enriched up- and down-regulated gene expression signatures in inherently trametinib sensitive and resistant primary patient-derived DIPG cultures. Heatmaps coloured according to the keys provided. Common up-regulated signatures between clones and primary cells are coloured red, common down-regulated signatures in blue. T1 *MEK2*_I115N, pink; T3 *MEK1*_I141S, purple;

T6, *MEK1*_K57N, red. (D) Barplot of normalised phospho-kinase array data for PDGFRB^{Y751} in ICR-B169 T6 (*MEK1*_K57N) cells treated with increasing concentrations of dasatinib. (E) Barplot of normalised phospho-kinase array data for AKT^{S473} in ICR-B169 T6 (*MEK1*_K57N) cells treated with increasing concentrations of dasatinib. (F) Barplot of normalised phospho-kinase array data for SRC^{Y419} in ICR-B169 T6 (*MEK1*_K57N) cells treated with increasing concentrations of dasatinib. (G) Barplot of normalised phospho-kinase array data for YES1^{Y426} in ICR-B169 T6 (*MEK1*_K57N) cells treated with increasing concentrations of dasatinib. (H) Dose-response validation curves for dasatinib tested against ICR-B181 cells *in vitro* grown in 3D (*PIK3R1*_N564D, blue) and 2D (*PIK3R1* wild-type, grey). (I) Dose-response curves for dasatinib tested against ICR-B184 cells *in vitro* grown in 3D (*NF1*_I1824S, blue) and 2D (*NF1* wild-type, grey). Concentration of compound is plotted on a log scale (x axis) against cell viability (y axis). Mean plus standard error are plotted from at least n=3 experiments. **p<0.01, *p<0.05, AUC t-test.

Supplementary Figure S7

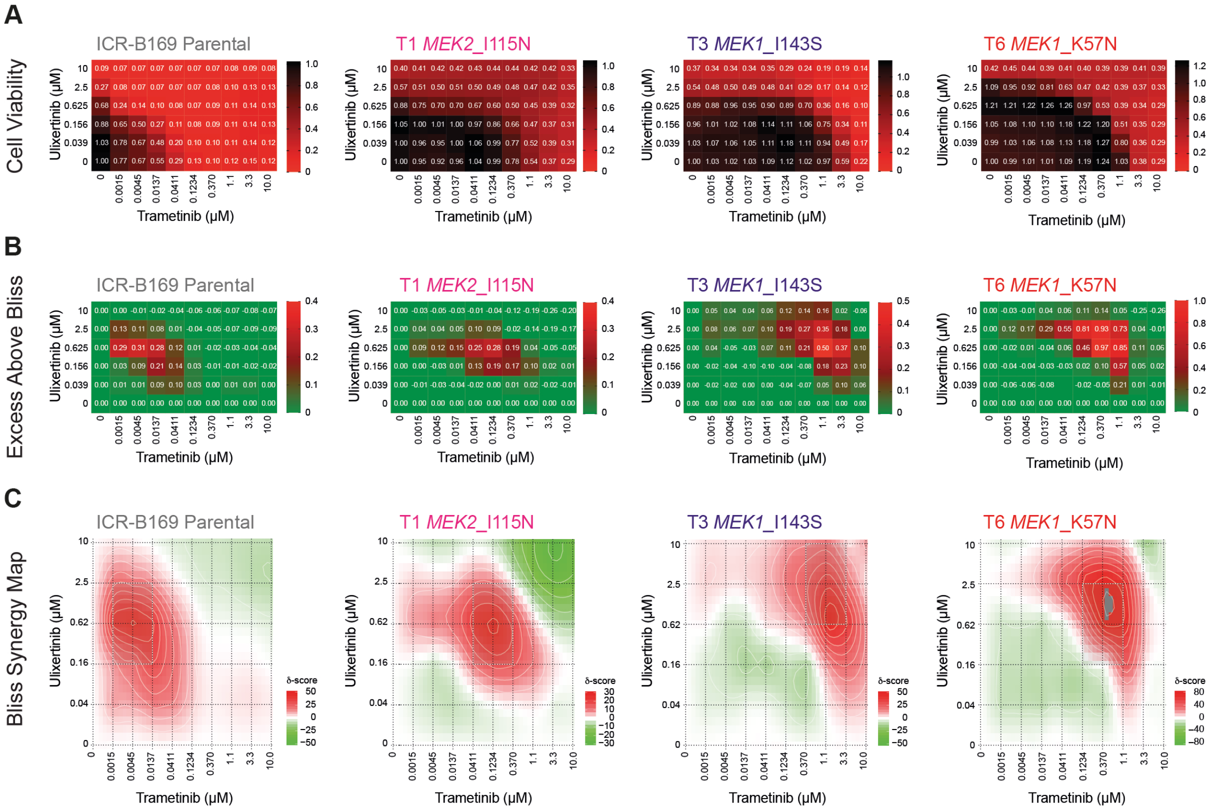

Supplementary Figure S7– *Synergy of combined ulixertinib and trametinib in BRAF_G469V-driven DIPG cells.* (A) Cell viability matrices for ICR-B169 parental (grey) and trametinib-resistant clones T1 (*MEK2*_I115N, pink), (T3 *MEK1*_I141S, purple) and T6 (*MEK1*_K57N, red), treated with distinct combinations of ulixertinib (y axes) and trametinib (x axes) ranging from 0-10 μM. A heatmap is overlaid to the proportions of viable cells remaining, coloured according to the key provided black (all cells) to red (no viable cells). (B) Excess above BLISS matrices for ICR-B169 parental and trametinib-resistant clones treated with distinct combinations of ulixertinib (y axes) and trametinib (x axes) ranging from 0-10 μM. A heatmap is overlaid to the excess score, coloured according to the key provided from red (enhanced effects) to green (no difference). (C) BLISS synergy maps for ICR-B169 parental and trametinib-resistant clones treated with distinct combinations of ulixertinib (y axes) and trametinib (x axes) ranging from 0-10 μM. The heatmap represents the δ score coloured according to the key provided from red (high degree of synergy) to green (antagonism).
